# A review on intrathecal administration of medications for leptomeningeal metastases in solid tumors

**DOI:** 10.3389/fphar.2025.1472945

**Published:** 2025-02-04

**Authors:** Xuemei Wang, Chi Yao, Li Quan, Junxiang Zhou

**Affiliations:** ^1^ Department of Clinical Pharmacy, Shifang People’s Hospital, North Sichuan Medical College, Deyang, China; ^2^ Department of Hepatobiliary Surgery, Shifang People’s Hospital, North Sichuan Medical College, Deyang, China; ^3^ Department of Pharmacy, Sichuan Clinical Research Center for Cancer, Sichuan Cancer Hospital & Institute, Sichuan Cancer Center, Afffliated Cancer Hospital of University of Electronic Science and Technology of China, Chengdu, China

**Keywords:** solid tumors, leptomeningeal disease, intrathecal therapy, chemotherapy drugs, immunotherapy drugs, targeted drugs, anti-tumor drugs

## Abstract

Leptomeningeal disease (LMD) is a particular mode of central metastasis in malignant tumors. It occurs when tumor cells infiltrate the subarachnoid space and cerebrospinal fluid (CSF), spreading throughout the central nervous system (CNS). LMD is a rare but devastating complication of malignant tumors. It can occur in various types of cancers, with lung and breast cancer being the most frequently associated. The treatment approach for LMD includes a combination of supportive care, surgery, chemotherapy, radiotherapy, targeted therapy, immunotherapy, and intrathecal (IT) therapy, among other modalities. Despite the challenges in determining the optimal treatment for LMD, IT therapy remains one of the primary therapeutic strategies. This therapy can directly circumvent the blood–brain barrier. Moreover, a low-dose medication can achieve a higher drug concentration in the CSF, resulting in better cytotoxic effects. Chemotherapy drugs such as methotrexate, cytarabine, and thiotepa have been widely studied as traditional IT therapies. In recent years, the advent of novel anti-tumor drugs has led to a growing number of agents being employed for IT administration in the treatment of malignant tumors with LMD. This article presents a comprehensive review of the current advancements in IT administration of chemotherapy, targeted, and immunotherapy drugs for the treatment of LMD in solid tumors. In addition, we also discuss the safety issues associated with IT therapy, summarize the advantages of IT administration of different types of anti-tumor drugs, and put forward some suggestions for reducing adverse reactions. It is hoped that future research will focus on exploring more potentially effective anti-tumor drugs for IT treatment, conducting in-depth pharmacokinetic studies, and developing long-acting and low-toxic IT administration regimens for the treatment of meningeal metastases.

## 1 Introduction

Leptomeningeal disease (LMD) of solid tumors is characterized by tumor cells invading the subarachnoid space and cerebrospinal fluid (CSF), leading to central metastasis of malignant tumors spreading throughout the central nervous system (CNS). It is a rare but fatal complication of malignant tumors. LMD can manifest across all types of malignant tumors. It is most prevalently observed in lung cancer, breast cancer, and melanoma (Thakkar et al., 2020). Approximately 5%–8% of patients with solid tumors can be associated with meningeal carcinomatosis (Carausu et al., 2021). The autopsy report shows that the incidence rate of many solid tumors exceeds 20% (Beauchesne, 2010; Carausu et al., 2021; Nguyen et al., 2021), indicating that the number of patients diagnosed with LMD is far lower than the actual incidence. LMD is more common in middle-aged and elderly people, with no significant gender differences. The median time from the primary tumor to the diagnosis of LMD is approximately 1–2 years, and the median overall survival (OS) is only 3–4 months, with a high mortality rate (Glantz et al., 1999a; Glantz et al., 1999b; Le Rhun et al., 2019). Hematological metastasis, lymphatic system metastasis, and CSF dissemination are its transmission pathways. The diagnosis of LMD requires the combination of the patient’s physical signs and symptoms with auxiliary examinations. In addition, the clinical manifestations are diverse and non-specific, with persistent headache as the main symptom of treatment (Wang et al., 2018). The overarching goals of treating LMD are twofold: first, to extend the survival of patients while ensuring a tolerable quality of life; second, to forestall or at least defer the progression of neurological deterioration. The LMD treatment plan encompasses supportive therapy, radiotherapy, chemotherapy, surgery, targeted therapy, immunotherapy, and intrathecal (IT) therapy (Pellerino et al., 2018). The specific treatment methods hinge on the histological characteristics, molecular expression, systemic disease progression, neurological function, prognosis, and other factors of the malignant tumor (Le Rhun et al., 2019; Thakkar et al., 2020).

**FIGURE 1:**
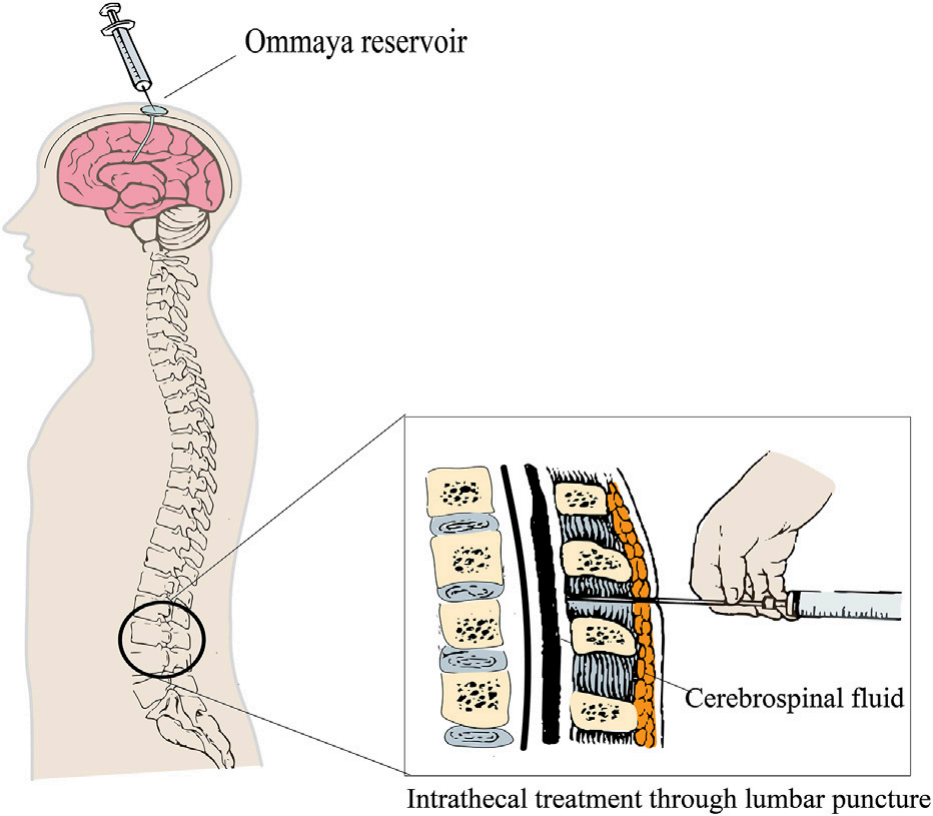
IT via ventricular catheter (Ommaya device) or lumbar puncture.

The central nervous system is frequently regarded as a “pharmacological sanctuary” since the majority of anti-cancer drugs are incapable of effectively penetrating the blood–brain barrier (BBB). IT therapy is a key treatment strategy for patients with LMD, offering the advantage of directly bypassing the blood–cerebrospinal fluid barrier to effectively target meningeal lesions. Additionally, given the significantly smaller volume of the CSF than that of plasma, low-dose administration achieves higher drug concentrations in the CSF, resulting in enhanced anti-tumor efficacy with reduced systemic toxicity. The European Society for Medical Oncology (ESMO) (Le Rhun et al., 2023) expert consensus recommends that IT therapy is suitable for most CSF-positive nodular or linear nodules (IA/C type) and patients with LMD with a high tumor cell load in the CSF. IT therapy can be directly administered into the lateral ventricle via subcutaneous fluid storage sacs and ventricular catheters (using an implanted Ommaya reservoir) or into the lumbar dural sac by lumbar puncture (Figure 1). Researchers suggest that drugs should be directly injected into the lateral ventricle through subcutaneous fluid storage sacs and ventricular catheters for IT therapy. Pharmacokinetic studies have shown that drugs can be evenly distributed across various parts of the CNS through CSF circulation, and the concentration can reach up to 10 times that of the same dose administered through lumbar puncture (Roguski et al., 2015; Le Rhun et al., 2017), avoiding the risk of failing to inject drugs into the CSF cavity (approximately 10%) (Shapiro et al., 1975; Glantz et al., 2010). The use of Ommaya intraventricular therapy resulted in significantly longer OS than that of lumbar puncture (9.2 vs. 4 months, p = 0.0006) (Montes de Oca Delgado et al., 2018). In addition, in patients with solid tumor LMD, the incidence of complications in the intraventricular fluid reservoir is lower (Wang et al., 2016). However, the Ommaya sac needs to be surgically inserted, and common complications include intracranial hemorrhage and catheter translocation and blockage. Overall, IT therapy with Ommaya is recommended as a priority for patients with LMD.

The most commonly used IT chemotherapy drugs for solid tumor LMD are methotrexate, thiotepa, and cytarabine (Le Rhun et al., 2023). However, no randomized clinical trials comparing any of these drugs with the optimal treatment for systemic diseases and the best supportive care have provided unequivocal evidence of a survival benefit in the treatment of solid tumor LMD (Boogerd et al., 2004). Nevertheless, occasional anecdotal responders and long-term survivors have been observed with IT chemotherapy. With continuous research and new explorations of diverse IT therapies for LMD, apart from chemotherapy drugs, targeted drugs and immunotherapy drugs also represent potential and effective pharmaceutical options. An increasing number of anti-tumor drugs are being recommended for the treatment of LMD. This article reviews the current research landscape and progress of IT anti-tumor drugs for solid tumors with LMD.

## 2 Standard IT therapeutic drugs

### 2.1 Cytotoxic drugs

#### 2.1.1 Methotrexate

As a classic anti-metabolic anti-tumor drug, methotrexate can effectively combat breast cancer and hematological malignancies. As early as 1977, researchers (Bleyer and Dedrick, 1977) recommended methotrexate to treat patients with LMD, extending the survival period from 4 to 9 months. This study also investigated the pharmacokinetics of IT methotrexate, measuring the concentration of methotrexate in the CSF of 76 patients. The concentration of methotrexate in the lumbar CSF decreased in a biphasic metabolic manner with half-lives of 4.5 and 8 h. It is not metabolized within the CSF. The drug is slowly absorbed through the choroid plexus, released into the systemic circulation, partially bound to serum albumin, and then excreted through the kidneys (Rubin et al., 1968).

Several small-scale prospective studies focused on IT methotrexate in patients with solid tumor LMD. These studies included participants ranging from several dozen to one hundred, and the primary tumors were predominantly lung and breast cancer, with CSF cytological remission rates reaching 20%–61%. These studies can validate the therapeutic efficacy of IT methotrexate (Grossman et al., 1993; Siegal et al., 1994; Glantz et al., 1998). IT methotrexate combined with radiotherapy emerges as a promising approach in clinical practice. Multiple studies included patients with solid tumor LMD who underwent IT methotrexate treatment combined with radiotherapy. Such a retrospective study (Wasserstrom et al., 1982) included 90 patients. Approximately half of the patients exhibited improvement in neurological symptoms, with a median OS of 5.8 months (1–29 months). In another retrospective study that enrolled 98 patients who underwent radiotherapy in combination with the standard-dose IT methotrexate, 41 patients were evaluated as having achieved a CR, while 12 patients attained a PR. The median OS ranged from 3 months among breast cancer patients to 8 months for lymphoma patients. Notably, the survival duration of patients who responded to methotrexate was significantly prolonged (Pfeffer et al., 1988). Similarly, a phase-II prospective single-arm clinical study included 59 patients (42 with lung cancer, 11 with breast cancer, and 6 with other) with adverse prognostic factors. The treatment consisted of IT methotrexate (12.5–15 mg) and dexamethasone (5 mg) administered once a week, combined with brain radiotherapy at a dose of 40Gy/20fr. The clinical effective rate was 86.4% (51/59), and the median OS was 6.5 months (0.4–36.7 months). This study is the first to preliminarily explore, through prospective clinical research, that the treatment regimen of IT methotrexate combined with regional radiotherapy is highly effective for solid tumor leptomeningeal carcinomatosis, which can improve the quality of life of patients, alleviate neurological symptoms, and prolong survival (Pan et al., 2016). The abovementioned studies indicate that the treatment mode of IT methotrexate and synchronous regional radiotherapy has significant benefits for patients with LMD.

A common dosage regimen for IT methotrexate therapy involves induction treatment with two injections per week for 4 weeks, followed by consolidation treatment with one injection per week for 4 weeks, and maintenance therapy with one injection per month until disease progression (Shapiro et al., 1975; Le Rhun et al., 2023). However, the dosages of IT methotrexate in the current literature are not consistent, with most doses varying between 10 and 15 mg. Hou et al. (2021) conducted a prospective single-arm clinical trial to investigate the clinical efficacy and safety of different doses of IT methotrexate in the treatment of cancerous meningitis. The study encompassed 53 patients, who were randomly assigned to a 15-mg dose group or a 10-mg dose group. The results showed that the median OS of the 15-mg dose group was 15.7 weeks, while that of the 10-mg dose group was 27.1 weeks, with no statistically significant difference (p = 0.116). Increasing the dosage of IT methotrexate appropriately did not prolong OS, but it was more effective in alleviating clinical symptoms and did not increase the incidence of adverse events. Continuous exposure to low concentrations of methotrexate throughout the body can lead to severe bone marrow suppression. Patients with renal insufficiency, pleural effusion or ascites, and abnormal CSF flow are at an increased risk of severe bone marrow suppression. IT methotrexate treatment can cause various neurological complications, including chemical meningitis, delayed white matter encephalopathy, acute encephalopathy, and transverse myelopathy (Boogerd et al., 2004; Stemmler et al., 2008; Pan et al., 2016). In order to minimize the risk of systemic methotrexate toxicity, all patients should be given oral folic acid (10 mg once, twice a day, for 3 days) simultaneously as a routine to reduce the systemic toxic reactions caused by methotrexate leaving the CSF and entering the bloodstream. Methotrexate, one of the classic IT chemotherapy drugs, has high efficacy in patients with solid tumor LMD. IT or combined radiation therapy can improve the quality of life of patients, prolong survival, and have good safety.

#### 2.1.2 Thiotepa

Thiotepa is an ethyleneimine alkylating agent that forms unstable ethylimine groups under physiological conditions and has strong cytotoxic effects. As a multifunctional alkylating agent, it inhibits nucleic acid synthesis, crosslinks with DNA, and is effective against ovarian, breast, bladder, and digestive tract cancers (Maanen et al., 2000). Thiotepa is a small-molecule, non-polar, highly lipid-soluble drug with good tissue infiltration. It has the shortest half-life among IT chemotherapy drugs. After IT therapy, it can quickly diffuse out of the CSF within 4 hours without being distributed to the CSF space or brain tissue, and it can be cleared through cerebral capillaries. A patient received IT 10 mg of thiotepa. The peak concentration of the CSF in the ventricles was >100 μg/mL, approximately 10 μg/mL after 2 hours, and approximately 1 μg/mL after 8 hours. The average clearance rate of the CSF was 1.8 mL/min, and the average systemic clearance rate was 518 mL/min (Strong et al., 1986).

The efficacy of IT thiotepa is not as clear as that of methotrexate. The typical dosage is 10 mg. The induction regimen entails two injections per week for a period of 4 weeks. Subsequently, the consolidation regimen involves one injection per week for 4 weeks. Thereafter, the maintenance regimen entails one injection per month until the disease progresses. If required, one injection per week can be implemented (Le Rhun et al., 2023). In most studies, thiotepa was administered at a dose of 10–15 mg per dose twice a week or continuously for 3 days per month (Chamberlain and Kormanik, 1997; Witham et al., 1999; Schrappe et al., 2000; Cho et al., 2015). As early as 1976, a phase I–II study on IT thiotepa was conducted in LMD patients with malignant tumors. The study involved 11 patients with meningeal leukemia, lymphoma, and ependymoma who received IT thiotepa at a dose of 1–10 mg/m^2^. Three patients achieved CR of the CNS symptoms, while five patients achieved PR. No hematological toxicity related to thiotepa was observed in this study, and neurotoxicity was limited to mild transient lower limb toxicity. This study preliminarily confirms the effectiveness and safety of IT thiotepa in patients with LMD (Gutin et al., 1976). Another multi-center randomized trial compared IT thiotepa and methotrexate in 59 patients with LMD, including breast cancer (48%), lung cancer (23%), lymphoma (19%), and other tumors (10%). It is important to mention that in this trial, the results showed that approximately one-third of patients in both groups achieved complete cytological clearance, and the median OS was similar between the groups (14 vs. 16 weeks) (Grossman et al., 1993). In order to further verify whether IT thiotepa has efficacy in breast cancer patients, a single-center retrospective study included 66 breast cancer LMD patients who were given IT thiotepa (10 mg) and methylprednisolone (40 mg) once every 2 weeks. This study reported comparable results; 16 out of the 36 evaluable patients had cytological remission, and the median OS was 4.5 months (Comte et al., 2013). This study confirmed that LMD patients with breast cancer had a good response to thiotepa. The selection of IT drugs following the failure of methotrexate treatment has always been a research focus. IT thiotepa appears to have good efficacy in LMD cases after methotrexate treatment failure. Cho et al. (2015) evaluated 398 patients with LMD of non-small-cell lung cancer (NSCLC) and breast cancer who were treated with IT therapy based on methotrexate. According to the standard treatment scheme, they received 10 mg of thiotepa twice a week for the rescue treatment of patients after the failure of methotrexate. Among the 30 patients who could be evaluated, 14 (47%) had cytological reactions to thiotepa in the CSF, and the median OS after the treatment with thiotepa was 19.4 weeks. This study confirmed that patients who failed methotrexate treatment could choose to use thiotepa for rescue treatment with good effectiveness.

IT administration of thiotepa is often well-tolerated and may cause systemic bone marrow suppression (Grossman et al., 1993). Neurotoxicity is limited to mild, transient lower-limb sensory abnormalities, and there have been occasional reports of spinal cord disease. As a classic IT chemotherapy drug such as methotrexate, thiotepa has good safety and effectiveness for LMD. At the same time, it is also a meaningful salvage treatment for patients with LMD who have progressed after methotrexate treatment.

#### 2.1.3 Cytarabine and cytarabine liposome

The structure of cytarabine is very similar to that of deoxycytidine, one of the components of DNA synthesis, which can interfere with DNA replication and kill tumor cells (Pace and Fabi, 2006). Cytarabine is mainly used for induction remission and maintenance therapy of acute non-lymphocytic leukemia in adults and children, and it is only effective for a small number of solid tumor patients. For solid tumors, such as lung and breast cancer, if the tumor has invaded the CSF or meninges, cytarabine can be used for IT therapy. The results were reported by Esteva et al. (2000) in seven patients with LMD from breast cancer, who were administered IT cytarabine three times in a single dose of 100 mg at 48-h intervals. The authors reported a mean (±SD) peak cytarabine concentration of 16.7 ± 6.3 mmol/L and a mean concentration of 0.77 ± 0.53 mmol/L after 6 h. The cytarabine concentration in the CSF decreased in a monophasic pattern with a concentration half-life of 1.45 ± 0.61 h. The accumulation of cytarabine in the CSF between the repeated injections was not observed, and cytarabine was not detectable in the plasma. The half-life of cytarabine in the CSF is less than 4 h, and it is completely cleared within 1–2 days (Fulton et al., 1982).

Cytarabine dosage is 25–100 mg, with an induction phase of two injections per week for 4 weeks, a consolidation phase of one injection per week for 4 weeks, and a maintenance phase of one injection per month until disease progression (Le Rhun et al., 2023). The researchers tested IT cytarabine in 32 patients with neoplastic meningitis from malignant gliomas and solid tumors (Fulton et al., 1982). The researchers administered cytarabine in two doses within just 1 week to the first group, which consisted of 20 patients, and 14 of these patients also received concurrent chemotherapy. All four patients exhibited clinical improvement. Among them, two patients remained alive for 8 weeks and 16 weeks, respectively, after the commencement of weekly cytarabine treatment. In another case, five patients demonstrated clinical improvement, and four of them were still alive without tumor recurrence at 26 weeks after the initiation of therapy. Although no distinct difference was observed between the two groups, it should be noted that these groups were not comparable in terms of prognostically significant clinical characteristics during diagnosis without recurrence. In the second group, three out of twelve patients were administered three consecutive doses. Shapiro et al. (2006) reported a series of 67 patients suffering from neoplastic meningitis caused by solid tumors, leukemia, and lymphomas. These patients received methotrexate or cytarabine alone or a combination of methotrexate and cytarabine through an intraventricular reservoir with or without CNS irradiation. Among these patients, 11 individuals underwent combination therapy with both drugs. Clinical improvement was observed in 58% of the patients with solid-tumor-related neoplastic meningitis. The median survival time for patients with breast cancer was 5 months. The above-mentioned research indicates that acute toxic effects associated with IT cytarabine included meningismus, nausea, vomiting, and myelosuppression.

Cytarabine liposome is a multi-vesicular system sustained-release liposome composition of cytarabine prepared using the sustained-release liposome platform. This nanostructure can encapsulate a large amount of drug and prolong the drug release time. It has been proven that the use of cytarabine liposome can delay the release of cytarabine, with a duration ranging from several days to several weeks and a longer half-life of over 80 h. Cytarabine liposome (50 mg) is administered intravenously every 2 weeks for LMD, with the number of IT does being only a quarter of that in conventional treatment (Jaeckle et al., 2001; Cole et al., 2003; Fusco et al., 2013). An open-label study (Phuphanich et al., 2007) investigated IT (50 mg) cytarabine liposome twice with a 14-day interval, analyzing the levels of cytarabine and metabolites in CSF and plasma samples. The results showed that the concentration range of free and encapsulated cytarabine in ventricular and lumbar CSF was 0.01–1,500 μg/mL, and it could be detected within 14 days. Occasionally, free cytarabine was detected in the plasma, and the concentrations of cytarabine metabolites were low in all samples.

To compare the efficacy of cytarabine liposome and methotrexate in patients with LMD, a randomized controlled trial was conducted, enrolling 61 patients with histologically confirmed cancer and positive CSF cytology. They were randomly divided into an IT cytarabine liposome group (31 patients) or a methotrexate group (30 patients). Patients received up to six doses of 50 mg cytarabine liposome or up to 16 doses of 10 mg methotrexate within 3 months. The trial results showed that in patients with LMD, cytarabine liposome was as effective as methotrexate and significantly increased the time of no progression in the nervous system while reducing the number of doses administered, providing convenience for patients (Glantz et al., 1999a). Another multi-center, randomized, controlled study also compared the benefits and safety of IT cytarabine liposome, methotrexate, and cytarabine in patients with solid tumor LMD (n = 103). The results showed that the benefits of cytarabine liposome and methotrexate in treating solid tumor LMD were comparable (Shapiro et al., 2006). These clinical trials encompassed a variety of cancer types, and the majority of the patients were non-primary cases. DEPOSEIN (NCT01645839) (Le Rhun et al., 2020) is a randomized open-label phase-III study that discusses the effectiveness and safety of IT cytarabine liposome combined with systemic therapy (n = 36) versus systemic therapy (n = 37) for patients with LMD from breast cancer at initial diagnosis. The study results indicate that the cytarabine liposome group significantly improved median PFS (3.8 vs. 2.2 months) and median OS (7.3 vs. 4 months) in patients with LMD. This study confirmed the role of IT cytarabine liposome in LMD patients with breast cancer. Cytarabine liposome, as a sustained-release liposome composition with a polycystic vesicle system, can encapsulate more drugs in its nanostructure, thereby reducing the administration time and increasing patient convenience. Clinical trials have also confirmed that for patients with solid tumor LMD, cytarabine liposome is as effective as methotrexate, which may be a good choice. Multiple studies have shown that the main adverse events of IT cytarabine liposome are headache and arachnoiditis. To prevent chemical meningitis caused by IT chemotherapy, each patient needs to be pretreated with dexamethasone.

#### 2.1.4 Etoposide

Etoposide acts on DNA topoisomerase II, forming a stable and reversible drug–enzyme–DNA complex that impedes DNA repair. Etoposide is mainly used for treating small-cell lung cancer (SCLC), malignant lymphoma, and malignant germ cell tumors (Issell and Crooke, 1979). The systemic administration of etoposide is effective in treating metastatic, recurrent, or refractory brain tumors, but its permeability to CSF is extremely low, almost not entering the CSF (<3%). Even after high-dose intravenous injection of etoposide, the CSF concentration does not exceed 0.1–0.5 μg/mL (Postmus et al., 1984; Kiya et al., 1992). The *in vitro* experimental results show that etoposide at concentrations ranging from 0.1 to 10 μg/mL exhibits cytotoxicity (Henwood and Brogden, 1990). IT etoposide can directly penetrate the BBB, and low-dose medication can lead to higher drug concentrations in the CSF. In a published case report (van der Gaast et al., 1992), IT (0.5 mg) etoposide was administered twice daily, with a 2-hour interval, for 5 days, followed by a second course of treatment 21 days later. Two hours after daily administration, the peak concentrations of the CSF in the ventricles were 3.6–5.2 μg/mL, and the trough concentrations of the CSF in the ventricles were 0.2–0.6 μg/mL. Another study reported that the peak concentration of etoposide in the CSF after IT administration exceeded that of intravenous infusion by more than 100 times. The steady-state distribution volume and total clearance rate showed significant individual differences, with a double exponential decrease. Etoposide was not detected in the plasma (Fleischhack et al., 2001).

Slavc et al. (2003) evaluated the long-term feasibility of IT etoposide in 11 children with disseminated non-hematological metastatic brain tumors. Two patients received IT mafosfamide before switching to intraventricular etoposide treatment, and nine patients received alternating IT etoposide and mafosfamide treatment. The dose of etoposide was 0.5 mg × 5 days, once every 3–6 weeks, for a total of 122 courses (1–29 courses/patient) until disease progression. The median time of etoposide treatment was 12 months (1–31 months), and the disease remission rate among the 11 patients was 54.5% (6/11). No long-term toxicity that could be attributed to IT therapy was observed. This study confirms that IT etoposide is both safe and effective for patients over 2 years old with LMD. In order to further validate the efficacy of IT etoposide in LMD of adult patients, another phase-II clinical trial (Chamberlain et al., 2006) was carried out, which included 27 adult patients (the median age was 55 years old); IT etoposide 0.5 mg per day was administered once every 5 days for 8 weeks to induce treatment. At the end of induction treatment, patients were evaluated through CSF cytology and neurological examination. Patients who had responded continued to receive etoposide treatment (every 4 weeks for 5 days) and undergo monthly evaluations. Among the 27 patients receiving etoposide treatment, 7 (26%) had stable or improved neurological status and 12 (44%) had no response to treatment. Among the patients who responded to treatment, the median PFS was 20 weeks (ranging from 8 to 20 weeks), and the 6-month PFS rate for neurological disease was 11%. For newly diagnosed LMD patients, the median OS is 10 weeks (4–52 weeks), and the survival probabilities at 3 months, 6 months, and 12 months are 30% (95% CI, 12%–47%), 11% (95% CI, 1%–21%), and 4% (95% CI, 0%–11%), respectively. During the treatment, five patients experienced transient chemical arachnoiditis, and no hematological toxicity related to etoposide was observed. The abovementioned report evaluated the efficacy of IT etoposide in the treatment of LMD, and patients demonstrated good tolerance to this treatment (Fleischhack et al., 2001; Slavc et al., 2003; Chamberlain et al., 2006). Based on the results of previous clinical trials on the efficacy of IT etoposide, Park (2015) first used IT etoposide to rescue a patient with NSCLC who failed first-line methotrexate treatment. The patient received 1 mg of etoposide intravenously once a week, and after 2 weeks of treatment, the brain lesion and clinical symptoms improved. However, the patient ultimately died due to liver metastasis. Unlike other studies, this study designed a weekly regimen of etoposide administration to rescue patients who failed methotrexate treatment, providing preliminary evidence for the feasibility of etoposide rescue therapy. In recent years, there has been relatively little research on IT etoposide. The safety of IT etoposide in children and young adults with refractory or recurrent malignant brain tumors was investigated (Pajtler et al., 2016). The research results showed that IT etoposide was well-tolerated, and the potential treatment-related adverse reactions included transient headache, epileptic seizures, nausea, and neuropsychological symptoms. No hematological side effects were observed.

#### 2.1.5 Topotecan

Topotecan is a topoisomerase I inhibitor with good therapeutic effects on various solid tumors in adults and children. The hydrolysis of topotecan lactone is pH-dependent, and the open carboxylate form as topoisomerase-I inhibitor has no activity. When topotecan is administered intravenously, approximately 30% reaches the CNS. After IT topotecan (0.4 mg) administration, the peak concentration of cerebroventricular CSF was 28 ± 11 μmol/L, and it was not detected in the plasma. The clearance of topotecan is mainly achieved through its conversion to inactive forms and CSF circulation, with only approximately 10% cleared through microvasculature (Stewart et al., 1994; Blaney et al., 2003; Glaberman et al., 2005).

A phase-I clinical trial (Blaney et al., 2003) enrolled 23 patients aged 3 years and above with tumor-associated LMD, who received IT topotecan to determine the optimal dose and dose-limiting toxicity. The study determined a maximum tolerable dose of 0.4 mg through a dose ramp-up test, and chemical subarachnoid inflammation was the dose-limiting toxicity that occurred within 24 h of administration, which was characterized by fever, nausea, vomiting, headache, and back pain. Among 23 assessable patients, six patients had stable disease, providing the optimal dosage for phase-II clinical trials. Another phase-II, open-label, non-randomized single-arm trial (Groves et al., 2008) included 62 patients with malignant LMD who received IT topotecan (0.4 mg) twice a week for 6 weeks. Primary cancers included breast cancer (n = 19), lung cancer (n = 13), CNS cancer (n = 14), and other cancers (n = 16). The estimated PFS rates at weeks 13 and 26 were 30% (20%–45%) and 19% (11%–34%), respectively. The median OS is 15 weeks (13–24 weeks). The most common side effect was chemical meningitis, which occurred in 32% of the patients (5% grade 3). IT topotecan was well-tolerated and had no obvious side effects. However, topotecan does not have significant advantages over other IT chemotherapy options. It can be combined with radiotherapy or systemic treatment to maximize the benefits for patients. Researchers treated two patients with recurrent or progressive CNS cancer with LMD using IT topotecan (0.2 mg/day) for 7 days in order to increase the exposure of topotecan in the CSF as much as possible (Tran et al., 2014). After administration, neither patient showed hematological toxicity or arachnoiditis that was grade 3 or above. Although this study is a case report, it offers a novel approach to individualized treatment options in clinical practice. In the latest study by Yamada et al. (2023), high-dose chemotherapy combined with stem cell rescue, followed by IT topotecan maintenance therapy, was used to treat six children with atypical rhabdomyoma. This treatment model successfully avoided whole-brain radiation therapy and prolonged their survival. IT topotecan maintenance therapy can improve CNS symptoms. The sample size of this study is small, and further large-scale research is needed to confirm this treatment model. IT topotecan is safe, with the most common adverse reactions being nausea, vomiting, headache, anorexia, and fever. Arachnoiditis is dose-limited toxicity, and no hematological toxicity was observed. Although topotecan does not have significant advantages over other IT chemotherapies, it can also be used as one of the drug choices for solid tumor LMD patients.

#### 2.1.6 Pemetrexed

Pemetrexed, as an anti-metabolic antitumor drug, is a first-line chemotherapy drug for NSCLC and exhibits good cytotoxicity. The incidence of NSCLC-LMD reaches as high as 5.0%–15.8% (Kwon et al., 2020). The pharmacokinetics of IT pemetrexed was investigated (Li et al., 2023). After IT 30 mg of pemetrexed was administered, a series of CSF samples were collected from eight patients, with 62.5% (5/8) reaching peak concentrations within 2–4 h, and the effective concentration level of pemetrexed in the CSF remained at an average of 93.44 μg/mL after 24 h of IT pemetrexed administration. The research results showed that pemetrexed exhibited a biphasic elimination mode in the CSF, while its concentration in the blood was extremely low.

To determine the efficacy of IT pemetrexed in lung adenocarcinoma patients with LMD who had undergone at least two previous treatments, a prospective phase-I study enrolled 23 lung adenocarcinoma LMD patients with failed multi-line treatment. According to the dose ramp-up test, IT pemetrexed at a dose of 30 mg was considered the optimal dose, with a median PFS of 6.3 months (ranging from 0.8 months to data not available (NA)) and a median OS of 9.5 months (ranging from 2.9 months to NA), respectively. In 2019, a study was reported on the use of IT pemetrexed for the salvage treatment of NSCLC-LMD, which was the world’s first phase-I clinical trial. The research results showed that the clinical effective rate of IT 10 mg pemetrexed was 31% (4/13), the disease control rate was 54% (7/13), and it had good tolerability. Subsequently, Pan et al. (2020) conducted a phase-I/II clinical study of IT pemetrexed and concurrent radiotherapy for solid tumor LMD. Based on the combination of affected field radiotherapy, the clinical effective rate of 10 mg pemetrexed was 68% (23/34). The median OS is 5.5 months (0.3–16.6 months). The research results suggest that the combination of IT pemetrexed and radiotherapy is effective for the treatment of LMD. In another prospective, single-center phase-II study conducted in the latest report (Fan et al., 2021), 30 patients with an epithelial growth factor receptor (EGFR) mutation and NSCLC-LMD were included and evaluated with IT pemetrexed and dexamethasone. The results showed a clinical efficacy rate of 84.6% and a median OS of 9 months, confirming that IT 50 mg pemetrexed has a high response rate and few adverse reactions in the treatment. These data are encouraging and offer new effective treatment methods for NSCLC-LMD patients. Multiple studies have demonstrated that pemetrexed exhibits good anti-tumor activity in the CSF without significant accumulation in the body. Bone marrow suppression and hepatotoxicity are the most prevalent adverse reactions (Pan et al., 2016; Geng et al., 2022). To reduce adverse reactions to pemetrexed, pre-treatment with oral folate, intramuscular injection of vitamin B12, and IT dexamethasone are equally important. Most studies use the classic dose escalation model to observe the tolerated dose and the relevant dose-limiting toxicity of IT pemetrexed. However, the sample size in these studies is relatively small. The dosages of pemetrexed vary significantly among different studies, and further larger sample studies are needed to further verify safety and effectiveness. Perhaps, after more clinical trials validating the safety and efficacy of IT pemetrexed in lung adenocarcinoma LMD patients, pemetrexed can replace methotrexate as a first-line treatment.

### 2.2 Targeted drugs

#### 2.2.1 Trastuzumab

Previous studies have shown that trastuzumab, whether as a single drug or in combination with chemotherapy, can delay the disease progression and improve the OS of breast cancer patients with human epidermal growth factor receptor 2 (HER-2) positivity (Kak et al., 2015; Malani et al., 2020). However, HER-2-positive breast cancer patients receiving systemic trastuzumab treatment often develop CNS metastasis, with approximately 20% of these cases occurring in LMD (Bendell et al., 2003). One of the reasons for stable or relieved CNS recurrence of systemic diseases may be the high molecular weight (145 kDa) of trastuzumab, which leads to its low CNS penetration rate. Research reported that when trastuzumab was administered intravenously on a weekly basis, there was a 300-fold difference in drug concentration between the patient’s serum and CSF (Pestalozzi and Brignoli, 2000). IT trastuzumab is a new treatment approach for HER-2-positive cancer, especially for patients with LMD from breast cancer. Multiple small-scale studies have shown that trastuzumab 25–150 mg has good stability when dissolved in physiological saline without preservatives (Laufman and Forsthoefel, 2001; Platini et al., 2006; Stemmler et al., 2006; Stemmler et al., 2008; Oliveira et al., 2011; Zagouri et al., 2013). The latest investigation shows that after IT (80 mg) trastuzumab administration, the distribution volume is 73 ± 48 mL, the CSF clearance rate is 14 ± 5 mL/h, and the apparent CSF half-life is 4.1 ± 3.0 h. The CSF half-life is much shorter than the 18–30 days half-life of trastuzumab intravenous administration. After repeated administration, the CSF concentration stabilizes, indicating that trastuzumab will not accumulate to toxic concentrations in the CSF (Kumthekar et al., 2023).

Trastuzumab was initially injected intrathecally for HER-2-overexpressing breast cancer patients. In these reports, patients who had received systemic treatment, IT chemotherapy, whole-brain radiotherapy, neurosurgery, and other treatment methods still did not experience relief. IT trastuzumab was used for rescue treatment. The literature has reported that the dose of IT trastuzumab ranges from 5–160 mg, either twice a week or once every 3 weeks. However, in most studies, the dose range is from 20 to 30 mg per week. Patients’ headaches, mental state, hemiplegia, ataxia, and other disorders were significantly improved. The duration of disease control with IT trastuzumab ranged from 39 days to over 72 months, and researchers observed no serious adverse reactions associated with trastuzumab (Laufman and Forsthoefel, 2001; Platini et al., 2006; Stemmler et al., 2006; Mir et al., 2008; Stemmler et al., 2008; Colozza et al., 2009; Ferrario et al., 2009; Oliveira et al., 2011; Smith et al., 2022). Some researchers believe that the effectiveness of IT trastuzumab seems to be dose-dependent. In the case that had been reported (Ferrario et al., 2009), patients who received IT trastuzumab 30 mg had better clinical benefits than those who received 20 mg. In another report (Mir et al., 2008), the IT dose of trastuzumab was increased to 50 mg in combination with 12 mg of thiotepa, which showed better efficacy than 20–30 mg. In a phase-I clinical trial (Bonneau et al., 2018), based on IT trastuzumab with a CSF concentration close to the conventional treatment plasma concentration (30 mg/L), a dose ramp-up test was conducted, and the maximum tolerable dose was determined to be 150 mg per week. In a large-scale phase-I/II multi-center study (Kumthekar et al., 2023), HER-2-positive histological cancer patients were included in the phase-I study, and the maximum tolerable dose for IT trastuzumab was determined to be 80 mg, twice a week, while ensuring both treatment effectiveness and safety. These studies indicate that the maximum tolerable overall dose per week for IT trastuzumab does not exceed 160 mg, rather than the traditional administration of 20–30 mg.

For patients with HER-2 overexpression in LMD associated with breast cancer, IT trastuzumab provides good benefits. Zagouri et al. (2013) retrospectively summarized and analyzed the effectiveness and safety of IT trastuzumab. A total of 13 articles were included, involving 17 patients with an average age of 48.2 years (38–66 years), and the average total dose was 399.8 mg (35–1,110 mg). With trastuzumab alone or in combination treatment, 88.2% of patients did not report serious adverse events, 68.8% of patients observed significant improvement in clinical symptoms, 31.2% of patients were stable or progressive, and the median OS was 13.5 months. The CNS-PFS is 7.5 months, and the longer CNS-PFS seems to be related to the improvement of clinical symptoms and CSF response. This retrospective study demonstrates the effectiveness and safety of IT trastuzumab. Based on a phase-I dose escalation test (Oberkampf et al., 2023), the study established the recommended dose of IT trastuzumab as 150 mg per week for the phase-II study. A total of 19 patients with LMD and HER-2-overexpressing breast cancer were included. All patients received at least one systemic anti-HER-2 treatment. After 8 weeks of IT trastuzumab administration, 74% (14/19) of patients had no neurological progress, the median LMD-related PFS was 5.9 months, and the median OS was 7.9 months. No adverse reactions of grade 3 or above were observed in all patients. Furthermore, a large-scale phase-I/II multi-center study (Kumthekar et al., 2023) included 23 HER-2-positive cancer patients who received IT trastuzumab 80 mg (twice a week for 4 weeks, then once a week for 4 weeks, followed by maintenance therapy every 1–2 weeks) for treatment. The partial response rate, disease stability rate, and disease progression rate were 19%, 50%, and 30%, respectively. At a median follow-up of 10.5 months, the median PFS and OS were 2.8 months and 10.5 months, respectively, indicating longer survival than that of the recognized historical control (median OS of approximately 3–4 months). In patients receiving treatment with IT trastuzumab, the most common side effects are headache, nausea, vomiting, and meningeal spasms, with only one case of grade-4 toxic reaction (arachnoiditis) found. However, in another meta-analysis (Lazaratos et al., 2023), the total survival period and PFS of IT trastuzumab (92 patients) and oral or intravenous HER-2 targeted therapy alternative route of administration (183 patients) in HER-2-positive breast cancer patients with LMD were compared. In univariate and multivariate analysis, the research results showed that there was no significant difference in OS and CNS-specific PFS between IT trastuzumab and oral or intravenous HER-2 targeted therapy. The study suggests that the reason for this result can be explained as follows: intravenous trastuzumab may reach the subarachnoid space at a sufficient concentration, effectively treating LMD in patients with trastuzumab-sensitive diseases. The patient had already received an intravenous injection of trastuzumab before IT therapy, which may have led to drug resistance. Even for patients receiving whole-brain radiotherapy, the concentration of trastuzumab in CSF is still one order of magnitude lower than the serum concentration (Stemmler et al., 2007; Steeg, 2021). Although this meta-analysis suggests that IT trastuzumab and HER-2 targeted therapy do not offer obvious advantages as an alternative route of administration, IT therapy can quickly alleviate patients’ neurological symptoms, and many clinical trials have shown that it has considerable efficacy and good tolerability for HER-2-positive breast cancer LMD patients. However, most of the enrolled clinical trials involve breast cancer with HER-2 positivity, which may not be applicable to the overall LMD population. Follow-up studies can include more HER-2-overexpressing solid tumor LMD patients. The most common adverse reactions of IT trastuzumab are headache, nausea, vomiting, and meningeal spasms.

### 2.3 Checkpoint inhibitors

#### 2.3.1 Nivolumab

Nivolumab is a programmed cell death protein 1 (PD-1) inhibitor approved for the treatment of NSCLC, head and neck squamous cell carcinoma, and gastric cancer, among others. Nivolumab specifically binds to PD-1 receptors on T cells, preventing signals from reaching T cells from malignant tumor cells and allowing T cells to function normally, thereby exerting anti-tumor effects. In multiple prospective clinical trials, it has been demonstrated to bring long-lasting benefits to patients with metastatic melanoma (Kluger et al., 2019; Tawbi et al., 2021; Wolchok et al., 2022). The intracranial objective response rate (ORR) of nivolumab was 20% in asymptomatic encephalomyelitis patients, 55% in the checkmate-204 trial (n = 101) (Long et al., 2018), and 51% in the ABC trial (n = 35) (Tawbi et al., 2021). In all these studies, the 3-year survival rate of responsive patients exceeded 90% (Tawbi et al., 2021). Therefore, nivolumab may have a significant benefit for patients with melanoma LMD.

For symptomatic patients with melanoma LMD, rapid relief of neurological symptoms is necessary. Compared with interleukin-2, anti PD-1 therapy has improved efficacy and safety. Therefore, experts hypothesized that IT anti PD-1 therapy for melanoma is safe and feasible, and a phase-I clinical study was conducted (Glitza Oliva et al., 2023). First, the toxicity of IT administration of anti-PD-1 was evaluated in an immunocompetent mouse model. Then, a first-ever human dose exploration study was designed using both IT and intravenous administration of anti-PD-1. A total of 25 melanoma LMD patients were included and treated with a dual-pronged approach, injecting nivolumab into two sites: IT 50 mg every 2 weeks to treat meningeal metastases and intravenous 240 mg to treat extra brain lesions. The median OS of these patients was 4.9 months, with 44% and 26% of patients surviving over 26 and 52 weeks, respectively. Notably, four patients survived for 74 weeks, 115 weeks, 136 weeks, and 143 weeks after receiving the first IT nivolumab dose, which far exceeded the expected survival period for patients with LMD after multiple treatments. The most common grade-1 or grade-2 adverse events in the study were nausea (36%), diarrhea (24%), decreased lymphocyte count (24%), elevated aspartate aminotransferase and/or alanine aminotransferase (24%), and rashes (24%). Patients who used IT nivolumab alone did not show grade 3 or higher toxicity. This is the first clinical study that has utilized two different routes of administration for nivolumab therapy in patients with LMD secondary to melanoma. This study provides a detailed explanation of the dose escalation, recommended IT dosage, safety profile, and preliminary efficacy of synchronous IT and intravenous administration of nivolumab in melanoma LMD patients, supporting the feasibility of prospective clinical trials in LMD melanoma patients. At the same time, this trial is the largest prospective clinical trial of IT immunotherapy among all types of cancers, and it is also the first systematic evaluation of the application of IT anti-PD-1 drugs. Although this study is focused on melanoma patients, it also holds reference significance for other types of tumors. This study represents an important step forward and a new treatment method for patients with LMD. Perhaps, in the future, IT therapy will no longer be limited to chemotherapy drugs such as methotrexate but could also be combined with PD-1 inhibitors.

## 3 Potential IT drugs

### 3.1 Targeted drugs

#### 3.1.1 Nimotuzumab

EGFR is a transmembrane glycoprotein with a molecular weight of 170 kD, and its intracellular region has special tyrosine kinase activity. *In vivo* and *in vitro* studies have shown that nimotuzumab can block the binding of EGFR and its ligand, and it has anti-angiogenic, anti-proliferative, and pro-apoptotic effects on tumors overexpressing EGFR (Crombet-Ramos et al., 2002). Nimotuzumab is suitable for combined treatment with radiotherapy for stage-III/IV nasopharyngeal carcinoma with positive EGFR expression. The efficacy of IT nimotuzumab in the treatment of solid tumor LMD may be related to the antibody-dependent cell-mediated cytotoxicity (ADCC) induced by nimotuzumab, and the effect of ADCC is usually observed 1–3 months after IT treatment. There are relatively few studies on IT nimotuzumab, with only two relevant reports. One of the reports (Ju et al., 2016a) included 20 patients with NSCLC-LMD who received IT nimotuzumab 50 mg per week. The average number of IT therapy sessions was 8.9 (range: 1–35), and the median OS after treatment was 5 months (2.4–7.6 months). The average CSF open pressure decreased from 270 mmH_2_O (202–338 mmH_2_O) before treatment to 140 mmH_2_O (127–153 mmH_2_O) (p < 0.001). All patients experienced LMD-related symptoms, especially headaches, in the nimotuzumab group. After IT nimotuzumab, all patients were relieved, and their quality of life improved. In the same year, another case report was published by Ju et al. (2016b), in which two patients were diagnosed with NSCLC-LMD. The first patient received IT nimotuzumab weekly at the dose of 25 mg, with an OS of 35 months after the diagnosis of LMD. The second patient received 50 mg, with an OS of 12.5 months. Both patients received systemic treatment before IT therapy. After 2–3 sessions of IT treatment, the neurological symptoms of both patients were significantly improved, and the opening pressure of CSF decreased. Neither of these reports mentioned the adverse reactions of IT nimotuzumab. There are relatively few studies on IT nimotuzumab, but it can also provide preliminary evidence of its effectiveness and safety. In the future, more research and larger sample sizes are needed for further verification.

#### 3.1.2 Bevacizumab

Bevacizumab is the first anti-tumor angiogenesis-targeting drug approved for marketing. It plays an anti-tumor role by specifically targeting vascular endothelial growth factors (VEGF), and it has definite efficacy in a variety of malignant tumors. At present, it has been approved for a variety of indications, including metastatic colorectal cancer and advanced/metastatic or recurrent NSCLC (Cao, 2008). Angiogenesis in solid tumor brain metastases occurs through multiple pathways, among which the VEGF-mediated pathway is the most important and extensively studied one. Research has confirmed that brain metastasis relies on VEGF, and the high expression of VEGF in brain metastases is associated with poor prognosis (Hanibuchi et al., 2014; Ebben and Ming, 2016). Basic studies have shown that bevacizumab can reach high concentrations in brain metastases, exerting its anti-angiogenic effect and significantly inhibiting the proliferation of brain metastases (Ilhan-Mutlu et al., 2016). Numerous studies have confirmed that bevacizumab can effectively inhibit the development of LMD in lung cancer patients without increasing the risk of cerebral hemorrhage (Besse et al., 2015; Gubens et al., 2018). The meta-analysis showed that in NSCLC patients with LMD, the ORR and disease control rate (DCR) of the anti-tumor treatment regimen with bevacizumab were significantly better than those of the current therapy. Patients treated with bevacizumab have better intracranial DCR and PFS than those treated extracranially (Liang et al., 2020). Bevacizumab can reduce the risk of new-onset LMD by 70%, indicating its potential to prevent brain metastasis, and the risk of intracranial hemorrhage caused by bevacizumab treatment is lower. The abovementioned research provides a theoretical basis for IT bevacizumab in the treatment of solid tumor LMD patients. There is relatively scant research on IT bevacizumab. In the experimental group, four New Zealand white rabbits were randomly selected to receive IT bevacizumab at a dose of 1.5 mg per week, while four rabbits in the control group received IT physiological saline for 4 weeks, aiming to explore the safety of IT bevacizumab. The experimental results indicate that all rabbits in the experimental group can tolerate repeated IT bevacizumab, and there is no alteration in the baseline neurological function state of the control group. In addition, no adverse reactions were observed. IT bevacizumab has no clinical or pathological neurotoxicity, and this neurotoxicity study provides safety data for IT bevacizumab (Brastianos et al., 2012). The first use of IT bevacizumab in humans was for the treatment of LMD in a glioblastoma patient (Holdaway et al., 2023). The 19-year-old female patient had a recurrence of glioblastoma in the thalamus and received two arterial infusions of bevacizumab. The disease remained relatively stable until it progressed in 2022. Magnetic resonance imaging then showed glioblastoma with LMD. Subsequently, five doses of IT bevacizumab at 25 mg, 37.5 mg, 50 mg, 50 mg, and 37.8 mg were administered, with a two-week interval between the first and fourth doses. As the patient had good tolerance and requested discharge, the interval between the fifth and fourth doses was only 1 day, thus reducing the dosage of the fifth dose. Throughout the treatment period, the patient had good tolerance and did not experience any serious adverse reactions or dose-limiting toxicity. After the first IT bevacizumab, the patient survived for 110 days. Due to the rapid progression of glioblastoma with LMD, the patient’s PFS could not be determined. Although the survival benefits of the patient could not be evaluated, the research team preliminarily verified the effectiveness and safety of IT bevacizumab through dose escalation experiments. IT bevacizumab may have good application prospects in solid tumor LMD.

### 3.2 Checkpoint inhibitors

#### 3.2.1 Pembrolizumab

Pembrolizumab, a humanized macromolecular monoclonal antibody, can selectively bind to PD-1 immune checkpoints on the surfaces of immune cells. It can accurately block the interaction between PD-1 and programmed death ligand 1 (PD-L1), relieve immune suppression mediated by the PD-1 pathway, promote T-cell proliferation and cytokine generation, and exert anti-tumor effects. It is approved for use in various solid tumors such as melanoma, NSCLC, esophageal cancer, and head and neck squamous cell carcinoma. Multiple studies have shown that systemic administration of pembrolizumab has good efficacy and safety for solid tumor LMD (Hendriks et al., 2019; Brastianos et al., 2020; Naidoo et al., 2021; Zheng et al., 2021). A phase-II clinical study included 13 patients with solid tumor LMD who received intravenous 200 mg pembrolizumab once every 3 weeks (Naidoo et al., 2021). The results showed that at 12 weeks, 38% of patients exhibited a CNS response (95% CI: 13.9%–68.4%). The median CNS-PFS and OS were 2.9 months (95% CI: 1.3-NA) and 4.9 months (95% CI: 3.7-NA), respectively. Only 15% of patients experienced treatment-related adverse events of grade 3 or higher.

Pembrolizumab has a limited amount of drug reaching the CSF through the BBB during systemic administration. A case report described a patient with triple-negative breast cancer who developed LMD following systemic administration of pembrolizumab and subsequently received IT pembrolizumab (Ensign et al., 2021). The patient was diagnosed with triple-negative breast cancer. After 2 years of systemic treatment, brain metastases occurred. Pembrolizumab was administered intravenously and combined with local radiotherapy for the brain. One year later, the patient developed LMD accompanied by cerebral edema, and two cycles of high-dose systemic methotrexate were given to control the neurological symptoms. However, imaging examinations indicated the progression of the CNS disease. Subsequently, 5 mg of pembrolizumab was injected intrathecally once every 3 weeks. During the first IT therapy, the patient did not have any adverse reactions. However, after the second administration, the patient experienced pain, weakness, nausea, and diplopia. Eventually, the patient died 9 weeks after receiving IT therapy. Coincidentally, the latest published study also reported the effects of IT pembrolizumab on a melanoma LMD patient (Dan et al., 2024). The patient was diagnosed with cutaneous melanoma, and genetic testing showed BRAF V600E mutation, PD-L1-positive expression, and a combined positive score of 10. After being diagnosed with LMD, the patient received whole-brain radiation therapy combined with targeted therapy and immunotherapy for 2 months, and the patient experienced disease progression. In May of the same year, the patient developed paraplegia, spinal hyperalgesia, nausea, vomiting, and headache, but the CSF cell pathology results were negative. The research group adjusted the anti-tumor regimen to 25 mg of pembrolizumab via IT injection and 175 mg via intravenous injection and continued with targeted therapy. No adverse events were reported after the first IT administration. The patient’s symptoms of nausea, vomiting, and headache were relieved, and muscle strength in both lower limbs improved. The patient then received the second IT pembrolizumab, but the symptoms did not improve significantly, and intermittent breathing and cardiac arrest occurred. Ultimately, the patient died of respiratory arrest 2 weeks after receiving IT pembrolizumab. The abovementioned reports suggest that it is not possible to draw a conclusion from case studies that IT pembrolizumab benefits patients with LMD, but it may help temporarily alleviate neurological symptoms. The findings of the abovementioned studies provide a new exploration direction for the administration of pembrolizumab; further studies using IT pembrolizumab are required before any therapeutic treatment decisions can be made concerning its efficacy and safety in the treatment of LMD.

### 3.3 Other immunotherapeutic drugs

#### 3.3.1 Chimeric antigen receptor natural killer

Numerous studies have shown that depletion of the natural killer (NK) cell population before tumor transplantation induces a more aggressive phenotype in metastatic tumors. The presence of a large number of tumor-infiltrating NK cells is associated with a good prognosis. NK cells play an important role in brain metastases and meningiomas, which have become the basis for the development of NK cell-based brain tumor therapies (Avril et al., 2012; Poli et al., 2013; Blaylock, 2015). Chimeric antigen receptor natural killer (CAR-NK) cell therapy is an innovative adoptive cell therapy that, through genetic engineering modification, connects antibodies or receptors that recognize target cell surface antigens with signaling molecules required to activate immune cells, thereby activating NK cells and allowing them to specifically attack tumor cells (Pan et al., 2022). As early as 2004, clinical trials were conducted using NK cells to treat recurrent malignant gliomas, and four out of nine patients who received treatment experienced tumor regression (Ishikawa et al., 2004). At present, there are no large-scale clinical trials reporting the effectiveness of NK cells in treating brain tumors or solid tumor LMD. Researchers first injected CAR-NK cells derived from umbilical cord blood intrathecally into T-cell acute lymphoblastic leukemia patients who experienced symptoms of CNS after receiving stem cell transplantation (Yuan et al., 2023). After a short period of infusion, the patient developed neurological symptoms including high fever, headache, nausea, vomiting, and spinal cord transection incontinence. However, the symptoms of the patient, including upper eyelid ptosis and blurred vision, were completely improved. After 9 months of IT CAR-NK cells, the patient’s bone marrow was completely chimeric with the donor. Although IT CAR-NK cells have certain neurotoxicity, adverse reactions can be reduced by increasing the dose of CAR-NK cells administered, increasing the number of IT, and improving the preparation process of CAR-NK cells. A phase-I clinical trial based on CAR-NK (NCT03383978) is currently underway, using IT NK-92/5.28.z CAR-NK to treat leptomeningeal spread caused by recurrent HER-2-positive glioblastoma. The abovementioned evidence suggests that IT CAR-NK cells may be a potentially feasible and effective option for treating tumor LMD in the future. For patients with solid tumor LMD, the BBB is a challenge faced by CAR-NK, including all macromolecular drugs. At present, changing the administration method, such as local delivery of NK cell therapeutic drugs through intraventricular and/or IT pathways, can increase drug concentration in the ventricular CSF, adjust NK cell distribution, and further kill tumor cells. However, further research and clinical trials are needed to verify the efficacy of IT NK cells.

## 4 Conclusions and future perspectives

IT anti-tumor drugs are a common method for treating LMD. IT chemotherapy drugs and targeted and immunotherapy drugs are summarized in Tables 1, 2, respectively. Based on current clinical data of antineoplastic drugs in patients with solid tumor LMD, methotrexate, thiotepa, cytarabine, and cytarabine liposomes are recommended by the ESMO (Le Rhun et al., 2023), while methotrexate, thiotepa, cytarabine, etoposide, topotecan, pemetrexed, trastuzumab, and nivolumab are endorsed by the National Comprehensive Cancer Network for the treatment of solid tumors with LMD (Central nervous system cancers V, 2024). Meanwhile, nimotuzumab, bevacizumab, and pembrolizumab have also been used by researchers for IT therapy. They may be potential IT drugs for patients with LMD from solid tumors. Although some efficacy has been achieved, the role of IT in the treatment of LMD patients with solid tumors is still controversial. Due to the short OS of patients with LMD, evaluating therapeutic efficacy is challenging. In addition, most immunotherapy and targeted drugs are reported as individual cases, lacking large-scale clinical trials and prospective evidence, and are less commonly used in clinical practice. Some studies have examined the effectiveness of combination IT therapy in solid cancers, such as IT administration of two anti-tumor drugs, IT therapy combined with radiotherapy, and IT therapy combined with systemic therapy (Martens et al., 2013; Pan et al., 2020), but most of the IT combination therapies we identified in the present review were case reports. Although these case reports suggested suitable efficacy and safety, these drug combinations should be validated in large, randomized, placebo-controlled clinical trials to enable clinicians to make accurate judgments concerning combination IT therapies for LMD.

**TABLE 1:** IT cytotoxic drugs for solid tumors with LMD.

Drug name	Conventional intravenous dosage	Intrathecal administration dosage	Intrathecal administration pre-treatment	Pharmacokinetic characteristics of intrathecal chemotherapy	Adverse reactions of intrathecal chemotherapy	Study type	Recommended guidelines for intrathecal administration	Reference
Methotrexate	40–60 mg/m^2^, once weekly	Induction therapy: 10–15 mg, twice weekly for 4 weeks Consolidation therapy: 10–15 mg, once a week, for 4 weeks Maintenance therapy: 15 mg, once a month	Folic acid (10 mg, twice a day, for 3 days) and IT or oral dexamethasone	The concentration of methotrexate in the lumbar CSF decreases in a biphasic metabolic manner, with half-lives of 4.5 and 8 h. The drug is slowly absorbed through the choroid plexus, released into the systemic circulation, partially bound to serum albumin, and excreted through the kidneys.	Chemical meningitis, delayed leukoencephalopathy, acute encephalopathy, and transverse myelopathy	Phase-II study, retrospective study	ESMO and NCCN	Rubin et al. (1968), Beauchesne (2010) Pan et al. (2016) Le Rhun et al. (2023)
Thiotepa	10 mg (0.2 mg/kg) per day for 5 days; adjusted to three times per week, with a total cumulative dose of 300 mg	Induction therapy: 10 mg, twice weekly for 4 weeks Consolidation therapy: 10 mg, once a week for 4 weeks Maintenance therapy: 10 mg, once a month	IT or oral dexamethasone	Thiotepa exhibits good tissue infiltration. It has the shortest half-life among IT chemotherapy drugs; it rapidly diffuses into the CSF within 4 h after IT, without distributing the CSF space or the brain parenchyma, and is cleared through cerebral capillaries.	Systemic bone marrow suppression, neurotoxicity limited to mild transient lower-limb sensory abnormalities, and spinal cord disease	Phase-I/II study, retrospective study	ESMO and NCCN	Gutin et al. (1976), Gutin et al. (1976), Strong et al. (1986), Beauchesne (2010), Le Rhun et al. (2023)
Cytarabine	100–200 mg/m^2^, administered once daily	Induction therapy: 25–100 mg, twice weekly for 4 weeks Consolidation therapy: 25–100 mg, once a week for 4 weeks Maintenance therapy: 25–100 mg, once a month	IT or oral dexamethasone	Accumulation of cytarabine in the CSF between the repeated injections was not observed, and cytarabine was not detectable in the plasma. The half-life of cytarabine in the CSF is less than 4 h, and it is completely cleared within 1–2 days.	Meningismus, nausea, vomiting, and myelosuppression	Phase-I/II study	ESMO and NCCN	Fulton et al. (1982), Le Rhun et al. (2023)
Cytarabine liposome	NA	50 mg, twice weekly for 8 weeks, followed by once monthly for a total duration of 24 weeks	IT or oral dexamethasone	The half-life is relatively long, exceeding 80 h IT 50 mg cytarabine liposome twice every 14 days; the concentration range of free and encapsulated cytarabine in the CSF of the ventricles and lumbar vertebrae is 0.01–1,500 μg/mL, which can be detected within 14 days after administration. Occasionally, free cytarabine is detected in the plasma, and the concentration of metabolites of cytarabine in the CSF and plasma is relatively low.	Headache and arachnoiditis	Phase-III/IV study	ESMO	Glantz et al. (1999a), Glantz et al. (1999b), Jaeckle et al. (2001), Shapiro et al. (2006), Phuphanich et al. (2007) Le Rhun et al. (2019)
Etoposide	60–100 mg/m^2^ for 3–5 days, either once every 3 weeks or once every 4 weeks	Induction therapy: 0.5 mg/day for 5 days, every other week for 8 weeks Consolidation therapy: 0.5 mg/day for 5 days, every other week for 4 weeks Maintenance therapy: 0.5 mg/day for 5 days, once a month	IT or oral dexamethasone	The peak concentration of etoposide in the CSF in the ventricles of the brain exceeds that of intravenous infusion by more than 100 times. The steady-state distribution volume and total clearance rate show significant individual differences, showing a double exponential decrease. No etoposide was detected in the plasma.	Transient headache, epileptic seizures, Ommaya sac infection, nausea, and neuropsychological symptoms	Phase-II study	NCCN	Fleischhack et al. (2001), Slavc et al. (2003), Chamberlain et al. (2006)
Topotecan	1.25 mg/m^2^, once daily for 5 days, repeated every 3 weeks	Induction therapy: 0.4 mg, twice weekly for 6 weeks Consolidation therapy: 0.4 mg, once weekly for 6 weeks Maintenance therapy: 0.4 mg, twice monthly for 4 months, followed by monthly administration thereafter	IT or oral dexamethasone	IT 0.4 mg topotecan, the peak concentration in the ventricles was 28 ± 11 μmol/L, and it was not detected in the plasma. The clearance was mainly achieved through its conversion into inactive forms and CSF circulation, with only approximately 10% clearance through microvasculature.	Nausea/vomiting, headache, anorexia, fever, and arachnoiditis (which indicates dose-limiting toxicity)	Phase-I study, phase-II study, and case report	NCCN	Blaney et al. (2003), Glaberman et al. (2005)
Pemetrexed	500 mg/m^2^, once every 3 weeks	Standard dosing: 50 mg, once every 3 weeks Induction therapy: 10 mg, twice weekly for 2 weeks Consolidation therapy: 10 mg, once weekly, with a total of no more than 8 doses	Take 400 μg of folic acid before IT therapy continuously for 5 days. Stop taking folic acid during and 1 month after discontinuing the therapy Inject 1,000 μg of vitamin B12 intramuscularly once a week before the first administration, and then administer it every 3 cycles thereafter IT or oral dexamethasone	IT 30 mg pemetrexed, the peak concentration reached 2–4 h. After 24 h of IT pemetrexed administration, the effective concentration level remained in the CSF, with an average value of 93.44 μg/mL. It showed a biphasic elimination pattern in the CSF and extremely low concentration in the blood.	Bone marrow suppression and hepatotoxicity	Phase-I study and phase-II study	NCCN	Pan et al. (2020), Fan et al. (2021), Li et al. (2023)

Abbreviations: IT, intrathecal; CSF, cerebrospinal fluid; ESMO, European Society for Medical Oncology; NCCN, National Comprehensive Cancer Network; NA, not available.

**TABLE 2:** IT targeted drugs and immunotherapy drugs for solid tumors with LMD.

Drug name	Conventional intravenous dosage	Intrathecal administration dosage	Adverse reactions of intrathecal targeted and immunotherapy drugs	Study type	Recommended guidelines for intrathecal administration	Reference
Trastuzumab	Two-week dosing regimen: an initial loading dose of 4 mg/kg, followed by a maintenance dose of 2 mg/kg Three-week dosing regimen: an initial loading dose of 8 mg/kg, followed by a maintenance dose of 6 mg/kg	80 mg–150 mg, twice weekly for 4 weeks, then once weekly for 4 weeks, followed by maintenance therapy every 1–2 weeks	Headache, nausea, vomiting, and meningeal spasms	Systematic review, phase-I study, phase-II study, phase-I/II study, and case report	NCCN	Zagouri et al. (2013), Kumthekar et al. (2023), Oberkampf et al. (2023), Lazaratos et al. (2023)
Nimotuzumab	100–400 mg, once a week, for a total of 8 weeks	50 mg, once a week	NA	Case report	—	Ju et al. (2016a), Ju et al. (2016b)
Bevacizumab	5 mg/kg, once every 3 weeks 7.5–15 mg/kg, once every 3 weeks	25–50 mg, once every 2 weeks	NA	Case report	—	Holdaway et al. (2023)
Nivolumab	3 mg/kg or 240 mg every 2 weeks or 480 mg every 4 weeks	50 mg, once every 2 weeks	Nausea, diarrhea, decreased lymphocyte count, elevated aspartate aminotransferase and/or alanine aminotransferase, and papules/rashes	Phase-I study	NCCN	Glitza Oliva et al. (2023)
Pembrolizumab	200 mg, once every 3 weeks, or 400 mg, once every 6 weeks	5 mg or 25 mg, once every 3 weeks	Pain, weakness, nausea, diplopia, intermittent breathing, and cardiac arrest	Case report	—	Dan et al. (2024)

Abbreviations: NCCN, National Comprehensive Cancer Network; NA, not available.

Notes: CAR-NK was not included in Table 2 due to the lack of recommended dosage and description of adverse reactions in the literature.

Some studies have proposed that when there are circulating tumor cells in the CSF, IT therapy should be considered first (Montes de Oca Delgado et al., 2018). The European Association of Neuro-Oncology and the ESMO recommend IT therapy when there is an absence of blood–cerebrospinal fluid barrier dysfunction (Le Rhun et al., 2023). In addition, the NCCN guidelines recommend that patients with LMD who have a Karnofsky Performance Status score greater than or equal to 60, no severe neurological deficits, a small systemic tumor burden, and can tolerate systemic therapy should receive IT therapy (Horbinski et al., 2023). Meanwhile, different types of IT anti-tumor drugs, such as chemotherapeutic, immunotherapeutic, and targeted drugs, have distinct advantages. IT chemotherapy drugs can directly deliver high concentrations of chemotherapeutic agents to the CSF, effectively killing tumor cells that have metastasized to the meninges. They have a good effect on controlling local tumors in the meninges and help alleviate symptoms such as headache and dizziness caused by meningeal metastasis. Therefore, IT chemotherapy drugs are effective for most solid tumors with LMD (Pfeffer et al., 1988). Targeted drugs can target specific targets of tumor cells with minimal damage to normal tissues. For example, for HER-2-positive breast cancer and glioblastoma with LMD, targeted drugs can more accurately inhibit tumor growth signaling pathways (Ferrario et al., 2009; Holdaway et al., 2023). Compared with traditional chemotherapy, due to its clear targeting, side effects are relatively easier to manage, and patients may have better tolerance to treatment, which can help improve their quality of life. Immunotherapy drugs can activate the patient’s own immune system, allowing immune cells to recognize and attack tumor cells that have metastasized to the meninges. In some patients who have melanoma and renal cell carcinoma with LMD, IT immunotherapy drugs can enhance the body’s anti-tumor immune response. After the immune system is activated, it may generate immune memory, inhibit tumor cell growth for a long time, potentially prolong the patient’s survival, and control tumor progression (Glitza Oliva et al., 2023).

However, the occurrence of adverse reactions has been repeatedly reported, and the safety of IT anti-tumor drugs has gained widespread attention in the medical community. Due to the BBB, most drugs injected within the sheath gather in the ventricles of the brain and do not circulate throughout the body. Therefore, the systemic toxicity of IT is relatively low. The most common complications of IT anti-tumor drugs are mainly related to their toxic effects (Beauchesne, 2010). Potential causes of adverse reactions are as follows: first, high concentrations of anti-tumor drugs exposed to the CSF have direct toxic effects on the CNS and induce changes in CSF biochemical levels (Rolf et al., 2006). Furthermore, after IT therapy, the outflow of CSF and the injection of medication result in an imbalance between intracranial pressure and spinal subarachnoid pressure, and brain tissue often flows into the lower pressure area. Therefore, patients often experience headaches and vomiting without fever, but anti-tumor drugs can damage white blood cells, leading to the release of endogenous pyrogens, which can also induce an increase in body temperature. In addition, the dura mater at the puncture site has a needle hole, and the CSF continuously flows out, causing a decrease in intracranial pressure and dilation of intracranial blood vessels, leading to headaches and vomiting. IT is usually performed simultaneously with systemic chemotherapy, which exacerbates the symptoms of adverse reactions. Finally, injecting impure drugs or introducing iodine from the skin into the CSF during puncture can trigger irritation due to puncture failure, bleeding, and other reasons. Increased secretion of the CSF can lead to elevated intracranial pressure, causing headaches, vomiting, fever, and more adverse effects. To reduce and prevent adverse reactions caused by IT therapy of anti-tumor therapy, attention should be paid to the following points: first, the operation of IT therapy needs to be standardized. It is necessary to strictly control the medication time interval, injection speed, and drug dosage. Second, attention should be paid to the potential risk of drug allergies, and it should be inquired whether the patient has experienced adverse reactions to IT anti-tumor drugs before choosing anti-tumor drugs with minimal toxic side effects, high safety, and sufficient evidence-based medicine as much as possible.

Although IT therapy for LMD has achieved some promising outcomes, it still faces many challenges. First, the optimal drug combinations and treatment regimens for IT therapy remain undetermined. Different drug combinations and treatment regimens may influence treatment outcomes and side effect profiles in various ways, necessitating further research and exploration. In addition, the long-term safety of IT therapy remains controversial. Although IT therapy can reduce the side effects caused by systemic chemotherapy, long-term use may cause damage to the nervous system and affect the quality of life. Moreover, clinical research on IT therapy for LMD also faces challenges. Addressing ethical issues related to patients, such as the risks of invasive procedures, complex risk explanations, therapeutic misconceptions, and strict ethical review standards, is particularly difficult. There is considerable heterogeneity among study participants, with patients often presenting with varying tumor types, stages, and treatment histories. The range of anti-tumor drugs available for IT therapy is limited, and their selection is frequently guided by empirical evidence or preliminary study findings, lacking precise screening mechanisms. Additional challenges include inaccurate evaluation criteria, insufficient safety monitoring, and difficulties in follow-up. In conclusion, although IT therapy for LMD has made some progress, it is still in the exploratory stage. In the future, it is necessary to further strengthen basic research and clinical research, explore more effective treatment regimens and drug combinations, improve treatment efficacy and safety, and ultimately provide greater hope for patients with LMD.

With the continuous progress of medical technology, the prospect of IT therapy for LMD is full of hope. On the one hand, an increasing number of new drugs are under development, and these drugs may have higher efficacy and fewer side effects. For example, some targeted and immunotherapy drugs have achieved remarkable results in other tumor fields and are expected to be applied to IT therapy for LMD in the future. On the other hand, the development of precision medicine and artificial intelligence can provide assistance for the individualized treatment of patients (Adam et al., 2020; Miao et al., 2020). Perhaps, in the near future, these two technologies can be utilized to assist in the design of IT administration regimens and drug screening. First, artificial intelligence is used to integrate data such as patient’s gene sequencing, pathology, and physical condition to accurately classify patients and screen out those suitable for IT treatment. Subsequently, it analyzes the multi-target characteristics of tumor cells, correlates them with the drug database, and selects matching multi-target drugs while also predicting their therapeutic efficacy and potential adverse reactions. During the treatment, the CSF indexes and symptom changes of the patients are continuously monitored. Artificial intelligence provides real-time feedback to dynamically fine-tune the drug dosage and injection frequency. The synergy between artificial intelligence and multi-target drugs can overcome the obstacles of individual differences and build a precise and flexible personalized treatment framework for different patients. The new generation of precision oncology utilizes computational models to personalize the prediction of patient responses to IT injections of anti-tumor drugs, enabling the determination of the optimal treatment plan. This approach holds great promise for improving the chances of successful recovery in patients.

LMD, characterized by a poor prognosis and complex treatment regimens, is one of the most severe complications of malignant tumors. It is thus recommended to integrate multidisciplinary treatment, which encompasses active systemic and local treatment approaches. IT treatment emerged as an important therapeutic option for patients with LMD. Although optimal IT therapy has not yet been established through clinical trials, it has demonstrated initial advantages in enhancing the efficacy and quality of life for solid tumor LMD patients. This involves not only classic IT drugs such as methotrexate and others but also attempts to administer other medications such as immunotherapeutic and targeted drugs. Future research is required to further evaluate the efficacy and safety of various IT drugs and determine the most appropriate approach and treatment combination.

## References

[B1] Adam G. Rampášek L. Safikhani Z. Smirnov P. Haibe-Kains B. Goldenberg A. (2020). Machine learning approaches to drug response prediction: challenges and recent progress. NPJ Precis. Oncol. 4, 19. 10.1038/s41698-020-0122-1 32566759 PMC7296033

[B2] Avril T. Vauleon E. Hamlat A. Saikali S. Etcheverry A. Delmas C. (2012). Human glioblastoma stem-like cells are more sensitive to allogeneic NK and T cell-mediated killing compared with serum-cultured glioblastoma cells. Brain Pathol. 22 (2), 159–174. 10.1111/j.1750-3639.2011.00515.x 21790828 PMC8029175

[B3] Beauchesne P. (2010). Intrathecal chemotherapy for treatment of leptomeningeal dissemination of metastatic tumours. Lancet Oncol. 11 (9), 871–879. 10.1016/s1470-2045(10)70034-6 20598636

[B4] Bendell J. C. Domchek S. M. Burstein H. J. Harris L. Younger J. Kuter I. (2003). Central nervous system metastases in women who receive trastuzumab-based therapy for metastatic breast carcinoma. Cancer 97 (12), 2972–2977. 10.1002/cncr.11436 12784331

[B5] Besse B. Le Moulec S. Mazières J. Senellart H. Barlesi F. Chouaid C. (2015). Bevacizumab in patients with nonsquamous non-small cell lung cancer and asymptomatic, untreated brain metastases (brain): a nonrandomized, phase II study. Clin. Cancer Res. 21 (8), 1896–1903. 10.1158/1078-0432.Ccr-14-2082 25614446

[B6] Blaney S. M. Heideman R. Berg S. Adamson P. Gillespie A. Geyer J. R. (2003). Phase I clinical trial of intrathecal topotecan in patients with neoplastic meningitis. J. Clin. Oncol. 21 (1), 143–147. 10.1200/jco.2003.04.053 12506183

[B7] Blaylock R. L. (2015). Cancer microenvironment, inflammation and cancer stem cells: a hypothesis for a paradigm change and new targets in cancer control. Surg. Neurol. Int. 6, 92. 10.4103/2152-7806.157890 26097771 PMC4455122

[B8] Bleyer W. A. Dedrick R. L. (1977). Clinical pharmacology of intrathecal methotrexate. I. Pharmacokinetics in nontoxic patients after lumbar injection. Cancer Treat. Rep. 61 (4), 703–708. 577895

[B9] Bonneau C. Paintaud G. Trédan O. Dubot C. Desvignes C. Dieras V. (2018). Phase I feasibility study for intrathecal administration of trastuzumab in patients with HER2 positive breast carcinomatous meningitis. Eur. J. Cancer 95, 75–84. 10.1016/j.ejca.2018.02.032 29635147

[B10] Boogerd W. van den Bent M. J. Koehler P. J. Heimans J. J. van der Sande J. J. Aaronson N. K. (2004). The relevance of intraventricular chemotherapy for leptomeningeal metastasis in breast cancer: a randomised study. Eur. J. Cancer 40 (18), 2726–2733. 10.1016/j.ejca.2004.08.012 15571954

[B11] Brastianos P. K. Brastianos H. C. Hsu W. Sciubba D. M. Kosztowski T. Tyler B. M. (2012). The toxicity of intrathecal bevacizumab in a rabbit model of leptomeningeal carcinomatosis. J. Neurooncol 106 (1), 81–88. 10.1007/s11060-011-0655-9 21789699

[B12] Brastianos P. K. Lee E. Q. Cohen J. V. Tolaney S. M. Lin N. U. Wang N. (2020). Single-arm, open-label phase 2 trial of pembrolizumab in patients with leptomeningeal carcinomatosis. Nat. Med. 26 (8), 1280–1284. 10.1038/s41591-020-0918-0 32483359

[B13] Cao Y. (2008). Molecular mechanisms and therapeutic development of angiogenesis inhibitors. Adv. Cancer Res. 100, 113–131. 10.1016/s0065-230x(08)00004-3 18620094

[B14] Carausu M. Carton M. Darlix A. Pasquier D. Leheurteur M. Debled M. (2021). Breast cancer patients treated with intrathecal therapy for leptomeningeal metastases in a large real-life database. ESMO Open 6 (3), 100150. 10.1016/j.esmoop.2021.100150 33984675 PMC8134714

[B15] Central nervous system cancers V (2024). NCCN clinical practice guidelines in oncology. Available at: https://www.nccn.org/professionals/physician_gls.

[B16] Chamberlain M. C. Kormanik P. R. (1997). Carcinomatous meningitis secondary to breast cancer: predictors of response to combined modality therapy. J. Neurooncol 35 (1), 55–64. 10.1023/a:1005803918194 9266441

[B17] Chamberlain M. C. Tsao-Wei D. D. Groshen S. (2006). Phase II trial of intracerebrospinal fluid etoposide in the treatment of neoplastic meningitis. Cancer 106 (9), 2021–2027. 10.1002/cncr.21828 16583432

[B18] Cho K. M. Kim Y. J. Kim S. H. Kim J. W. Lee J. O. Han J. H. (2015). Salvage treatment with intracerebrospinal fluid thiotepa in patients with leptomeningeal metastasis after failure of methotrexate-based treatment. Anticancer Res. 35 (10), 5631–5638. 26408736

[B19] Cole B. F. Glantz M. J. Jaeckle K. A. Chamberlain M. C. Mackowiak J. I. (2003). Quality-of-life-adjusted survival comparison of sustained-release cytosine arabinoside versus intrathecal methotrexate for treatment of solid tumor neoplastic meningitis. Cancer 97 (12), 3053–3060. 10.1002/cncr.11449 12784341

[B20] Colozza M. Minenza E. Gori S. Fenocchio D. Paolucci C. Aristei C. (2009). Extended survival of a HER-2-positive metastatic breast cancer patient with brain metastases also treated with intrathecal trastuzumab. Cancer Chemother. Pharmacol. 63 (6), 1157–1159. 10.1007/s00280-008-0859-7 18987856

[B21] Comte A. Jdid W. Guilhaume M. N. Kriegel I. Piperno-Neumann S. Dieras V. (2013). Survival of breast cancer patients with meningeal carcinomatosis treated by intrathecal thiotepa. J. Neurooncol 115 (3), 445–452. 10.1007/s11060-013-1244-x 24043602

[B22] Crombet-Ramos T. Rak J. Pérez R. Viloria-Petit A. (2002). Antiproliferative, antiangiogenic and proapoptotic activity of h-R3: a humanized anti-EGFR antibody. Int. J. Cancer 101 (6), 567–575. 10.1002/ijc.10647 12237899

[B23] Dan X. Huang M. Sun Z. Chu X. Shi X. Chen Y. (2024). Case report: concurrent intrathecal and intravenous pembrolizumab for metastatic melanoma with leptomeningeal disease. Front. Oncol. 14, 1344829. 10.3389/fonc.2024.1344829 38665955 PMC11043509

[B24] Ebben J. D. Ming Y. (2016). Brain metastasis in lung cancer: building a molecular and systems-level understanding to improve outcomes. Int. J. Biochem. Cell. Biol. 78, 288–296. 10.1016/j.biocel.2016.07.025 27474492 PMC6020150

[B25] Ensign S. P. F. Yancey E. Anderson K. S. Mrugala M. M. (2021). Safety and feasibility of intrathecal pembrolizumab infusion in refractory triple negative breast cancer with leptomeningeal disease: a case report. Curr. Problems Cancer Case Rep. 4, 100103. 10.1016/j.cpccr.2021.100103

[B26] Esteva F. J. Soh L. T. Holmes F. A. Plunkett W. Meyers C. A. Forman A. D. (2000). Phase II trial and pharmacokinetic evaluation of cytosine arabinoside for leptomeningeal metastases from breast cancer. Cancer Chemother. Pharmacol. 46 (5), 382–386. 10.1007/s002800000173 11127942

[B27] Fan C. Zhao Q. Li L. Shen W. Du Y. Teng C. (2021). Efficacy and safety of intrathecal pemetrexed combined with dexamethasone for treating tyrosine kinase inhibitor-failed leptomeningeal metastases from EGFR-mutant NSCLC-a prospective, open-label, single-arm phase 1/2 clinical trial (unique identifier: ChiCTR1800016615). J. Thorac. Oncol. 16 (8), 1359–1368. 10.1016/j.jtho.2021.04.018 33989780

[B28] Ferrario C. Davidson A. Bouganim N. Aloyz R. Panasci L. C. (2009). Intrathecal trastuzumab and thiotepa for leptomeningeal spread of breast cancer. Ann. Oncol. 20 (4), 792–795. 10.1093/annonc/mdp019 19223574

[B29] Fleischhack G. Reif S. Hasan C. Jaehde U. Hettmer S. Bode U. (2001). Feasibility of intraventricular administration of etoposide in patients with metastatic brain tumours. Br. J. Cancer 84 (11), 1453–1459. 10.1054/bjoc.2001.1841 11384092 PMC2363656

[B30] Fulton D. S. Levin V. A. Gutin P. H. Edwards M. S. Seager M. L. Stewart J. (1982). Intrathecal cytosine arabinoside for the treatment of meningeal metastases from malignant brain tumors and systemic tumors. Cancer Chemother. Pharmacol. 8 (3), 285–291. 10.1007/bf00254052 6897022

[B31] Fusco J. P. Castañón E. Carranza O. E. Zubiri L. Martín P. Espinós J. (2013). Neurological and cytological response as potential early predictors of time-to-progression and overall survival in patients with leptomeningeal carcinomatosis treated with intrathecal liposomal cytarabine: a retrospective cohort study. J. Neurooncol 115 (3), 429–435. 10.1007/s11060-013-1241-0 24037499

[B32] Geng D. Guo Q. Huang S. Zhang H. Guo S. Li X. (2022). A retrospective study of intrathecal pemetrexed combined with systemic therapy for leptomeningeal metastasis of lung cancer. Technol. Cancer Res. Treat. 21, 15330338221078429. 10.1177/15330338221078429 35289201 PMC8928347

[B33] Glaberman U. Rabinowitz I. Verschraegen C. F. (2005). Alternative administration of camptothecin analogues. Expert Opin. Drug Deliv. 2 (2), 323–333. 10.1517/17425247.2.2.323 16296757

[B34] Glantz M. J. Cole B. F. Recht L. Akerley W. Mills P. Saris S. (1998). High-dose intravenous methotrexate for patients with nonleukemic leptomeningeal cancer: is intrathecal chemotherapy necessary? J. Clin. Oncol. 16 (4), 1561–1567. 10.1200/jco.1998.16.4.1561 9552066

[B35] Glantz M. J. Jaeckle K. A. Chamberlain M. C. Phuphanich S. Recht L. Swinnen L. J. (1999a). A randomized controlled trial comparing intrathecal sustained-release cytarabine (DepoCyt) to intrathecal methotrexate in patients with neoplastic meningitis from solid tumors. Clin. Cancer Res. 5 (11), 3394–3402. 10589750

[B36] Glantz M. J. LaFollette S. Jaeckle K. A. Shapiro W. Swinnen L. Rozental J. R. (1999b). Randomized trial of a slow-release versus a standard formulation of cytarabine for the intrathecal treatment of lymphomatous meningitis. J. Clin. Oncol. 17 (10), 3110–3116. 10.1200/jco.1999.17.10.3110 10506606

[B37] Glantz M. J. Van Horn A. Fisher R. Chamberlain M. C. (2010). Route of intracerebrospinal fluid chemotherapy administration and efficacy of therapy in neoplastic meningitis. Cancer 116 (8), 1947–1952. 10.1002/cncr.24921 20151421

[B38] Glitza Oliva I. C. Ferguson S. D. Bassett R. Jr. Foster A. P. John I. Hennegan T. D. (2023). Concurrent intrathecal and intravenous nivolumab in leptomeningeal disease: phase 1 trial interim results. Nat. Med. 29 (4), 898–905. 10.1038/s41591-022-02170-x 36997799 PMC10115650

[B39] Grossman S. A. Finkelstein D. M. Ruckdeschel J. C. Trump D. L. Moynihan T. Ettinger D. S. (1993). Randomized prospective comparison of intraventricular methotrexate and thiotepa in patients with previously untreated neoplastic meningitis. Eastern Cooperative Oncology Group. J. Clin. Oncol. 11 (3), 561–569. 10.1200/jco.1993.11.3.561 8445432

[B40] Groves M. D. Glantz M. J. Chamberlain M. C. Baumgartner K. E. Conrad C. A. Hsu S. (2008). A multicenter phase II trial of intrathecal topotecan in patients with meningeal malignancies. Neuro Oncol. 10 (2), 208–215. 10.1215/15228517-2007-059 18316473 PMC2613823

[B41] Gubens M. A. Chuang J. C. Akerley W. Langer C. J. Clément-Duchêne C. San Pedro-Salcedo M. (2018). A pooled analysis of advanced nonsquamous non-small cell lung cancer patients with stable treated brain metastases in two phase II trials receiving bevacizumab and pemetrexed as second-line therapy. J. Thorac. Dis. 10 (1), 219–227. 10.21037/jtd.2017.12.30 29600052 PMC5863135

[B42] Gutin P. H. Weiss H. D. Wiernik P. H. Walker M. D. (1976). Intrathecal N, N', N″-triethylenethiophosphoramide [thio-TEPA (NSC 6396)] in the treatment of malignant meningeal disease: phase I-II study. Cancer 38 (4), 1471–1475. 10.1002/1097-0142(197610)38:4<1471::aid-cncr2820380404>3.0.co;2-0 825215

[B43] Hanibuchi M. Kim S. J. Fidler I. J. Nishioka Y. (2014). The molecular biology of lung cancer brain metastasis: an overview of current comprehensions and future perspectives. J. Med. Invest. 61 (3-4), 241–253. 10.2152/jmi.61.241 25264041

[B44] Hendriks L. E. L. Bootsma G. Mourlanette J. Henon C. Mezquita L. Ferrara R. (2019). Survival of patients with non-small cell lung cancer having leptomeningeal metastases treated with immune checkpoint inhibitors. Eur. J. Cancer 116, 182–189. 10.1016/j.ejca.2019.05.019 31203193

[B45] Henwood J. M. Brogden R. N. (1990). Etoposide. A review of its pharmacodynamic and pharmacokinetic properties, and therapeutic potential in combination chemotherapy of cancer. Drugs 39 (3), 438–490. 10.2165/00003495-199039030-00008 2184009

[B46] Holdaway M. Ablyazova F. Huda S. D'Amico R. S. Wong T. Shani D. (2023). First in-human intrathecal delivery of bevacizumab for leptomeningeal spread from recurrent glioblastoma: rationale for a dose escalation trial. J. Neurooncol 164 (1), 231–237. 10.1007/s11060-023-04412-5 37548850

[B47] Horbinski C. Nabors L. B. Portnow J. Baehring J. Bhatia A. Bloch O. (2023). NCCN Guidelines® insights: central nervous system cancers, version 2.2022. J. Natl. Compr. Canc Netw. 21 (1), 12–20. 10.6004/jnccn.2023.0002 36634606

[B48] Hou L. Han W. Jin J. Chen X. Zou Y. Yan L. (2021). Clinical efficacy and safety of different doses of intrathecal methotrexate in the treatment of leptomeningeal carcinomatosis: a prospective and single-arm study. Jpn. J. Clin. Oncol. 51 (12), 1715–1722. 10.1093/jjco/hyab155 34585252

[B49] Ilhan-Mutlu A. Osswald M. Liao Y. Gömmel M. Reck M. Miles D. (2016). Bevacizumab prevents brain metastases formation in lung adenocarcinoma. Mol. Cancer Ther. 15 (4), 702–710. 10.1158/1535-7163.Mct-15-0582 26809491

[B50] Ishikawa E. Tsuboi K. Saijo K. Harada H. Takano S. Nose T. (2004). Autologous natural killer cell therapy for human recurrent malignant glioma. Anticancer Res. 24 (3b), 1861–1871. 15274367

[B51] Issell B. F. Crooke S. T. (1979). Etoposide (VP-16-213). Cancer Treat. Rev. 6 (2), 107–124. 10.1016/s0305-7372(79)80045-6 385138

[B52] Jaeckle K. A. Phuphanich S. Bent M. J. Aiken R. Batchelor T. Campbell T. (2001). Intrathecal treatment of neoplastic meningitis due to breast cancer with a slow-release formulation of cytarabine. Br. J. Cancer 84 (2), 157–163. 10.1054/bjoc.2000.1574 11161370 PMC2363714

[B53] Ju Y. Sun S. Wang J. Jiao S. (2016b). Prolonged overall survival of patients with leptomeningeal carcinomatosis from nonsmall cell lung cancer. J. Cancer Res. Ther. 12 (Suppl. ment), 126–129. 10.4103/0973-1482.191638 27721269

[B54] Ju Y. Wang J. Sun S. Jiao S. (2016a). Nimotuzumab treatment and outcome analysis in patients with leptomeningeal metastasis from nonsmall cell lung cancer. J. Cancer Res. Ther. 12 (Suppl. ment), C181–c185. 10.4103/0973-1482.200596 28230014

[B55] Kak M. Nanda R. Ramsdale E. E. Lukas R. V. (2015). Treatment of leptomeningeal carcinomatosis: current challenges and future opportunities. J. Clin. Neurosci. 22 (4), 632–637. 10.1016/j.jocn.2014.10.022 25677875

[B56] Kiya K. Uozumi T. Ogasawara H. Sugiyama K. Hotta T. Mikami T. (1992). Penetration of etoposide into human malignant brain tumors after intravenous and oral administration. Cancer Chemother. Pharmacol. 29 (5), 339–342. 10.1007/bf00686001 1312906

[B57] Kluger H. M. Chiang V. Mahajan A. Zito C. R. Sznol M. Tran T. (2019). Long-term survival of patients with melanoma with active brain metastases treated with pembrolizumab on a phase II trial. J. Clin. Oncol. 37 (1), 52–60. 10.1200/jco.18.00204 30407895 PMC6354772

[B58] Kumthekar P. U. Avram M. J. Lassman A. B. Lin N. U. Lee E. Grimm S. A. (2023). A phase I/II study of intrathecal trastuzumab in human epidermal growth factor receptor 2-positive (HER2-positive) cancer with leptomeningeal metastases: safety, efficacy, and cerebrospinal fluid pharmacokinetics. Neuro Oncol. 25 (3), 557–565. 10.1093/neuonc/noac195 35948282 PMC10013631

[B59] Kwon B. S. Cho Y. H. Yoon S. K. Lee D. H. Kim S. W. Kwon D. H. (2020). Impact of clinicopathologic features on leptomeningeal metastasis from lung adenocarcinoma and treatment efficacy with epidermal growth factor receptor tyrosine kinase inhibitor. Thorac. Cancer 11 (2), 436–442. 10.1111/1759-7714.13296 31910497 PMC6996974

[B60] Laufman L. R. Forsthoefel K. F. (2001). Use of intrathecal trastuzumab in a patient with carcinomatous meningitis. Clin. Breast Cancer 2 (3), 235. 10.1016/s1526-8209(11)70419-0 11899418

[B61] Lazaratos A. M. Maritan S. M. Quaiattini A. Darlix A. Ratosa I. Ferraro E. (2023). Intrathecal trastuzumab versus alternate routes of delivery for HER2-targeted therapies in patients with HER2+ breast cancer leptomeningeal metastases. Breast 69, 451–468. 10.1016/j.breast.2023.04.008 37156650 PMC10300571

[B62] Le Rhun E. Preusser M. van den Bent M. Andratschke N. Weller M. (2019). How we treat patients with leptomeningeal metastases. ESMO Open 4 (Suppl. 2), e000507. 10.1136/esmoopen-2019-000507 31231573 PMC6555600

[B63] Le Rhun E. Wallet J. Mailliez A. Le Deley M. C. Rodrigues I. Boulanger T. (2020). Intrathecal liposomal cytarabine plus systemic therapy versus systemic chemotherapy alone for newly diagnosed leptomeningeal metastasis from breast cancer. Neuro Oncol. 22 (4), 524–538. 10.1093/neuonc/noz201 31637444 PMC7158648

[B64] Le Rhun E. Weller M. Brandsma D. Van den Bent M. de Azambuja E. Henriksson R. (2017). EANO-ESMO Clinical Practice Guidelines for diagnosis, treatment and follow-up of patients with leptomeningeal metastasis from solid tumours. Ann. Oncol. 28 (Suppl. l_4), iv84–iv99. 10.1093/annonc/mdx221 28881917

[B65] Le Rhun E. Weller M. van den Bent M. Brandsma D. Furtner J. Rudà R. (2023). Leptomeningeal metastasis from solid tumours: EANO-ESMO Clinical Practice Guideline for diagnosis, treatment and follow-up. ESMO Open 8 (5), 101624. 10.1016/j.esmoop.2023.101624 37863528 PMC10619142

[B66] Li H. Zheng S. Lin Y. Yu T. Xie Y. Jiang C. (2023). Safety, pharmacokinetic and clinical activity of intrathecal chemotherapy with pemetrexed via the Ommaya reservoir for leptomeningeal metastases from lung adenocarcinoma: a prospective phase I study. Clin. Lung Cancer 24 (2), e94–e104. 10.1016/j.cllc.2022.11.011 36588048

[B67] Liang P. Wang Y. D. Wei Z. M. Deng Q. J. Xu T. Liu J. (2020). Bevacizumab for non-small cell lung cancer patients with brain metastasis: a meta-analysis. Open Med. (Wars) 15 (1), 589–597. 10.1515/med-2020-0192 33313410 PMC7706125

[B68] Long G. V. Atkinson V. Lo S. Sandhu S. Guminski A. D. Brown M. P. (2018). Combination nivolumab and ipilimumab or nivolumab alone in melanoma brain metastases: a multicentre randomised phase 2 study. Lancet Oncol. 19 (5), 672–681. 10.1016/s1470-2045(18)30139-6 29602646

[B69] Maanen M. J. Smeets C. J. Beijnen J. H. (2000). Chemistry, pharmacology and pharmacokinetics of N,N',N″ -triethylenethiophosphoramide (ThioTEPA). Cancer Treat. Rev. 26 (4), 257–268. 10.1053/ctrv.2000.0170 10913381

[B70] Malani R. Fleisher M. Kumthekar P. Lin X. Omuro A. Groves M. D. (2020). Cerebrospinal fluid circulating tumor cells as a quantifiable measurement of leptomeningeal metastases in patients with HER2 positive cancer. J. Neurooncol 148 (3), 599–606. 10.1007/s11060-020-03555-z 32506369 PMC7438284

[B71] Martens J. Venuturumilli P. Corbets L. Bestul D. (2013). Rapid clinical and radiographic improvement after intrathecal trastuzumab and methotrexate in a patient with HER-2 positive leptomeningeal metastases. Acta Oncol. 52 (1), 175–178. 10.3109/0284186x.2012.689857 22655969

[B72] Miao R. Chen H. H. Dang Q. Xia L. Y. Yang Z. Y. He M. F. (2020). Beyond the limitation of targeted therapy: improve the application of targeted drugs combining genomic data with machine learning. Pharmacol. Res. 159, 104932. 10.1016/j.phrs.2020.104932 32473309

[B73] Mir O. Ropert S. Alexandre J. Lemare F. Goldwasser F. (2008). High-dose intrathecal trastuzumab for leptomeningeal metastases secondary to HER-2 overexpressing breast cancer. Ann. Oncol. 19 (11), 1978–1980. 10.1093/annonc/mdn654 18845838

[B74] Montes de Oca Delgado M. Cacho Díaz B. Santos Zambrano J. Guerrero Juárez V. López Martínez M. S. Castro Martínez E. (2018). The comparative treatment of intraventricular chemotherapy by Ommaya reservoir vs. Lumbar puncture in patients with leptomeningeal carcinomatosis. Front. Oncol. 8, 509. 10.3389/fonc.2018.00509 30524956 PMC6256195

[B75] Naidoo J. Schreck K. C. Fu W. Hu C. Carvajal-Gonzalez A. Connolly R. M. (2021). Pembrolizumab for patients with leptomeningeal metastasis from solid tumors: efficacy, safety, and cerebrospinal fluid biomarkers. J. Immunother. Cancer 9 (8), e002473. 10.1136/jitc-2021-002473 34380662 PMC8359453

[B76] Nguyen T. K. Nguyen E. K. Soliman H. (2021). An overview of leptomeningeal disease. Ann. Palliat. Med. 10 (1), 909–922. 10.21037/apm-20-973 32921068

[B77] Oberkampf F. Gutierrez M. Trabelsi Grati O. Le Rhun É. Trédan O. Turbiez I. (2023). Phase II study of intrathecal administration of trastuzumab in patients with HER2-positive breast cancer with leptomeningeal metastasis. Neuro Oncol. 25 (2), 365–374. 10.1093/neuonc/noac180 35868630 PMC9925703

[B78] Oliveira M. Braga S. Passos-Coelho J. L. Fonseca R. Oliveira J. (2011). Complete response in HER2+ leptomeningeal carcinomatosis from breast cancer with intrathecal trastuzumab. Breast Cancer Res. Treat. 127 (3), 841–844. 10.1007/s10549-011-1417-2 21369716

[B79] Pace A. Fabi A. (2006). Chemotherapy in neoplastic meningitis. Crit. Rev. oncology/hematology 60 (3), 194–200. 10.1016/j.critrevonc.2006.06.013 16949298

[B80] Pajtler K. W. Tippelt S. Siegler N. Reichling S. Zimmermann M. Mikasch R. (2016). Intraventricular etoposide safety and toxicity profile in children and young adults with refractory or recurrent malignant brain tumors. J. Neurooncol 128 (3), 463–471. 10.1007/s11060-016-2133-x 27147083

[B81] Pan K. Farrukh H. Chittepu V. Xu H. Pan C. X. Zhu Z. (2022). CAR race to cancer immunotherapy: from CAR T, CAR NK to CAR macrophage therapy. J. Exp. Clin. Cancer Res. 41 (1), 119. 10.1186/s13046-022-02327-z 35361234 PMC8969382

[B82] Pan Z. Yang G. He H. Cui J. Li W. Yuan T. (2020). Intrathecal pemetrexed combined with involved-field radiotherapy as a first-line intra-CSF therapy for leptomeningeal metastases from solid tumors: a phase I/II study. Ther. Adv. Med. Oncol. 12, 1758835920937953. 10.1177/1758835920937953 32733606 PMC7370561

[B83] Pan Z. Yang G. He H. Zhao G. Yuan T. Li Y. (2016). Concurrent radiotherapy and intrathecal methotrexate for treating leptomeningeal metastasis from solid tumors with adverse prognostic factors: a prospective and single-arm study. Int. J. Cancer 139 (8), 1864–1872. 10.1002/ijc.30214 27243238 PMC5096248

[B84] Park M. J. (2015). Prolonged response of meningeal carcinomatosis from non-small cell lung cancer to salvage intrathecal etoposide subsequent to failure of first-line methotrexate: a case report and literature review. Am. J. Case Rep. 16, 224–227. 10.12659/ajcr.894061 25879815 PMC4407681

[B85] Pellerino A. Bertero L. Rudà R. Soffietti R. (2018). Neoplastic meningitis in solid tumors: from diagnosis to personalized treatments. Ther. Adv. Neurol. Disord. 11, 1756286418759618. 10.1177/1756286418759618 29535794 PMC5844521

[B86] Pestalozzi B. C. Brignoli S. (2000). Trastuzumab in CSF. J. Clin. Oncol. 18 (11), 2349–2351. 10.1200/jco.2000.18.11.2349 10829059

[B87] Pfeffer M. R. Wygoda M. Siegal T. (1988). Leptomeningeal metastases-treatment results in 98 consecutive patients. Isr. J. Med. Sci. 24 (9-10), 611–618. 3144516

[B88] Phuphanich S. Maria B. Braeckman R. Chamberlain M. (2007). A pharmacokinetic study of intra-CSF administered encapsulated cytarabine (DepoCyt) for the treatment of neoplastic meningitis in patients with leukemia, lymphoma, or solid tumors as part of a phase III study. J. Neurooncol 81 (2), 201–208. 10.1007/s11060-006-9218-x 16941075

[B89] Platini C. Long J. Walter S. (2006). Meningeal carcinomatosis from breast cancer treated with intrathecal trastuzumab. Lancet Oncol. 7 (9), 778–780. 10.1016/s1470-2045(06)70864-6 16945774

[B90] Poli A. Wang J. Domingues O. Planagumà J. Yan T. Rygh C. B. (2013). Targeting glioblastoma with NK cells and mAb against NG2/CSPG4 prolongs animal survival. Oncotarget 4 (9), 1527–1546. 10.18632/oncotarget.1291 24127551 PMC3824525

[B91] Postmus P. E. Holthuis J. J. Haaxma-Reiche H. Mulder N. H. Vencken L. M. van Oort W. J. (1984). Penetration of VP 16-213 into cerebrospinal fluid after high-dose intravenous administration. J. Clin. Oncol. 2 (3), 215–220. 10.1200/jco.1984.2.3.215 6321690

[B92] Roguski M. Rughani A. Lin C. T. Cushing D. A. Florman J. E. Wu J. K. (2015). Survival following Ommaya reservoir placement for neoplastic meningitis. J. Clin. Neurosci. 22 (9), 1467–1472. 10.1016/j.jocn.2015.04.003 26115896

[B93] Rolf N. Boehm H. Kaindl A. M. Lauterbach I. Suttorp M. (2006). Acute ascending motoric paraplegia following intrathecal chemotherapy for treatment of acute lymphoblastic leukemia in children: case reports and review of the literature. Klin. Padiatr 218 (6), 350–354. 10.1055/s-2006-942276 17080338

[B94] Rubin R. Owens E. Rall D. (1968). Transport of methotrexate by the choroid plexus. Cancer Res. 28 (4), 689–694. 5649058

[B95] Schrappe M. Reiter A. Zimmermann M. Harbott J. Ludwig W. D. Henze G. (2000). Long-term results of four consecutive trials in childhood ALL performed by the ALL-BFM study group from 1981 to 1995. Berlin-Frankfurt-Münster. Berlin-Frankfurt-Münster. Leuk. 14 (12), 2205–2222. 10.1038/sj.leu.2401973 11187912

[B96] Shapiro W. R. Schmid M. Glantz M. Miller J. J. (2006). A randomized phase III/IV study to determine benefit and safety of cytarabine liposome injection for treatment of neoplastic meningitis. J. Clin. Oncol. 24, 1528. 10.1200/jco.2006.24.18_suppl.1528

[B97] Shapiro W. R. Young D. F. Mehta B. M. (1975). Methotrexate: distribution in cerebrospinal fluid after intravenous, ventricular and lumbar injections. N. Engl. J. Med. 293 (4), 161–166. 10.1056/nejm197507242930402 806016

[B98] Siegal T. Lossos A. Pfeffer M. R. (1994). Leptomeningeal metastases: analysis of 31 patients with sustained off-therapy response following combined-modality therapy. Neurology 44 (8), 1463–1469. 10.1212/wnl.44.8.1463 8058150

[B99] Slavc I. Schuller E. Falger J. Günes M. Pillwein K. Czech T. (2003). Feasibility of long-term intraventricular therapy with mafosfamide (n = 26) and etoposide (n = 11): experience in 26 children with disseminated malignant brain tumors. J. Neurooncol 64 (3), 239–247. 10.1023/a:1025633704071 14558599

[B100] Smith P. D. Bhenderu L. S. Kommuri S. Fleener E. E. Hoover J. M. (2022). Treatment of leptomeningeal carcinomatosis following treatment of cerebellar metastasis of HER2+ (human epidermal growth factor receptor 2 positive) breast cancer: case report and review of literature. Cureus 14 (4), e24008. 10.7759/cureus.24008 35547416 PMC9090228

[B101] Steeg P. S. (2021). The blood-tumour barrier in cancer biology and therapy. Nat. Rev. Clin. Oncol. 18 (11), 696–714. 10.1038/s41571-021-00529-6 34253912

[B102] Stemmler H. J. Mengele K. Schmitt M. Harbeck N. Laessig D. Herrmann K. A. (2008). Intrathecal trastuzumab (Herceptin) and methotrexate for meningeal carcinomatosis in HER2-overexpressing metastatic breast cancer: a case report. Anticancer Drugs 19 (8), 832–836. 10.1097/CAD.0b013e32830b58b0 18690096

[B103] Stemmler H. J. Schmitt M. Harbeck N. Willems A. Bernhard H. Lässig D. (2006). Application of intrathecal trastuzumab (Herceptintrade mark) for treatment of meningeal carcinomatosis in HER2-overexpressing metastatic breast cancer. Oncol. Rep. 15 (5), 1373–1377. 10.3892/or.15.5.1373 16596213

[B104] Stemmler H. J. Schmitt M. Willems A. Bernhard H. Harbeck N. Heinemann V. (2007). Ratio of trastuzumab levels in serum and cerebrospinal fluid is altered in HER2-positive breast cancer patients with brain metastases and impairment of blood-brain barrier. Anticancer Drugs 18 (1), 23–28. 10.1097/01.cad.0000236313.50833.ee 17159499

[B105] Stewart C. F. Baker S. D. Heideman R. L. Jones D. Crom W. R. Pratt C. B. (1994). Clinical pharmacodynamics of continuous infusion topotecan in children: systemic exposure predicts hematologic toxicity. J. Clin. Oncol. 12 (9), 1946–1954. 10.1200/jco.1994.12.9.1946 8083716

[B106] Strong J. M. Collins J. M. Lester C. Poplack D. G. (1986). Pharmacokinetics of intraventricular and intravenous N,N',N''-triethylenethiophosphoramide (thiotepa) in rhesus monkeys and humans. Cancer Res. 46 (12 Pt 1), 6101–6104. 3096555

[B107] Tawbi H. A. Forsyth P. A. Hodi F. S. Algazi A. P. Hamid O. Lao C. D. (2021). Long-term outcomes of patients with active melanoma brain metastases treated with combination nivolumab plus ipilimumab (CheckMate 204): final results of an open-label, multicentre, phase 2 study. Lancet Oncol. 22 (12), 1692–1704. 10.1016/s1470-2045(21)00545-3 34774225 PMC9328029

[B108] Thakkar J. P. Kumthekar P. Dixit K. S. Stupp R. Lukas R. V. (2020). Leptomeningeal metastasis from solid tumors. J. Neurol. Sci. 411, 116706. 10.1016/j.jns.2020.116706 32007755

[B109] Tran H. C. Gardner S. Weiner H. L. Liebes L. F. Finlay J. L. (2014). Pilot study assessing a seven-day continuous intrathecal topotecan infusion for recurrent or progressive leptomeningeal metastatic cancer. J. Oncol. Pharm. Pract. 20 (3), 229–232. 10.1177/1078155213494940 23929729

[B110] van der Gaast A. Sonneveld P. Mans D. R. Splinter T. A. (1992). Intrathecal administration of etoposide in the treatment of malignant meningitis: feasibility and pharmacokinetic data. Cancer Chemother. Pharmacol. 29 (4), 335–337. 10.1007/bf00685957 1311219

[B111] Wang A. Tenner M. S. Tobias M. E. Mohan A. Kim D. Tandon A. (2016). A novel approach using electromagnetic neuronavigation and a flexible neuroendoscope for placement of Ommaya reservoirs. World Neurosurg. 96, 195–201. 10.1016/j.wneu.2016.08.127 27609447

[B112] Wang N. Bertalan M. S. Brastianos P. K. (2018). Leptomeningeal metastasis from systemic cancer: review and update on management. Cancer 124 (1), 21–35. 10.1002/cncr.30911 29165794 PMC7418844

[B113] Wasserstrom W. R. Glass J. P. Posner J. B. (1982). Diagnosis and treatment of leptomeningeal metastases from solid tumors: experience with 90 patients. Cancer 49 (4), 759–772. 10.1002/1097-0142(19820215)49:4<759::aid-cncr2820490427>3.0.co;2-7 6895713

[B114] Witham T. F. Fukui M. B. Meltzer C. C. Burns R. Kondziolka D. Bozik M. E. (1999). Survival of patients with high grade glioma treated with intrathecal thiotriethylenephosphoramide for ependymal or leptomeningeal gliomatosis. Cancer 86 (7), 1347–1353. 10.1002/(sici)1097-0142(19991001)86:7<1347::aid-cncr34>3.0.co;2-m 10506724

[B115] Wolchok J. D. Chiarion-Sileni V. Gonzalez R. Grob J. J. Rutkowski P. Lao C. D. (2022). Long-term outcomes with nivolumab plus ipilimumab or nivolumab alone versus ipilimumab in patients with advanced melanoma. J. Clin. Oncol. 40 (2), 127–137. 10.1200/jco.21.02229 34818112 PMC8718224

[B116] Yamada A. Kinoshita M. Kamimura S. Jinnouchi T. Azuma M. Yamashita S. (2023). Novel strategy involving high-dose chemotherapy with stem cell rescue followed by intrathecal topotecan maintenance therapy without whole-brain irradiation for atypical teratoid/rhabdoid tumors. Pediatr. Hematol. Oncol. 40 (7), 629–642. 10.1080/08880018.2023.2220734 37519026

[B117] Yuan J. Wang F. Ren H. (2023). Intrathecal CAR-NK cells infusion for isolated CNS relapse after allogeneic stem cell transplantation: case report. Stem Cell. Res. Ther. 14 (1), 44. 10.1186/s13287-023-03272-0 36941630 PMC10029298

[B118] Zagouri F. Sergentanis T. N. Bartsch R. Berghoff A. S. Chrysikos D. de Azambuja E. (2013). Intrathecal administration of trastuzumab for the treatment of meningeal carcinomatosis in HER2-positive metastatic breast cancer: a systematic review and pooled analysis. Breast Cancer Res. Treat. 139 (1), 13–22. 10.1007/s10549-013-2525-y 23588955

[B119] Zheng M. M. Tu H. Y. Yang J. J. Zhang X. C. Zhou Q. Xu C. R. (2021). Clinical outcomes of non-small cell lung cancer patients with leptomeningeal metastases after immune checkpoint inhibitor treatments. Eur. J. Cancer 150, 23–30. 10.1016/j.ejca.2021.03.037 33882375

